# Wastewater mining for circular potassium fertilisers: A critical scoping review of technologies, product formulations, and up-scaling challenges

**DOI:** 10.1016/j.ese.2026.100777

**Published:** 2026-10-08

**Authors:** Cora Eichholz, Heidrun Steinmetz

**Affiliations:** Department of Resource-Efficient Wastewater Treatment, RPTU Kaiserslautern-Landau, Paul-Ehrlich-Straße 14, Kaiserslautern, 67663, Germany

**Keywords:** Resource recovery, Potassium recycling, Circular bioeconomy, Bio-based fertiliser

## Abstract

Potassium (K) is an essential plant macronutrient and an irreplaceable resource for global food production. Today, agricultural potassium comes almost entirely from mined potash, an energy-intensive practice linked to severe ecological pressures—such as the salinisation of vulnerable river systems—and heavy dependence on a few exporting nations. Recovering potassium from wastewater offers a tangible path toward circular nutrient management, yet research on K recovery has largely been overshadowed by efforts focused on nitrogen and phosphorus. This scoping review critically evaluates 110 peer-reviewed publications using the Joanna Briggs Institute framework combined with citation network analysis to assess where the field genuinely stands. Our analysis reveals that research remains predominantly in its infancy: roughly 34% of studies rely on synthetic solutions in simplified lab-scale setups, with investigations centring on K-struvite precipitation (approximately 43%), electrodialysis, and ion exchange. These processes face acute operational hurdles under real-world conditions, severe competition from counter-ions (NH_4_^+^ and Na^+^), scaling challenges, and a lack of long-term sorbent regeneration and stability data. Crucially, we show that the wastewater stream choice fundamentally dictates the feasible fertiliser formulation. High-strength, nutrient-rich streams—such as source-separated urine (900–2440 mg K L^−1^) and liquid digestate (1494–2440 mg K L^−1^)—are naturally suited for producing solid compound fertilisers (predominantly K-struvite), whereas low-concentration municipal WWTP effluents (approximately 25 mg K L^−1^) offer a more favourable stoichiometric balance for isolating liquid or single-nutrient fertilisers (e.g., KCl for precision hydroponics). However, before these secondary fertilisers can enter the agricultural market, critical gaps must be resolved regarding product purity, compliance with the European Union Fertilising Products Regulation (particularly heavy metal and organic micropollutant thresholds), and synergistic multi-stage process design. Ultimately, moving potassium recovery from laboratory curiosity into existing wastewater infrastructure demands rigorous pilot testing with dynamic, real effluents alongside holistic life-cycle assessments.

## Highlights

• Scoping review of 110 studies reveals wastewater K recovery remains largely confined to lab scale.
• K-struvite and electrodialysis face severe counterion (NH_4_^+^ and Na^+^) interference and scaling in real matrices.
• Wastewater characteristics dictate products: concentrated streams yield solid compounds, dilute effluents yield liquid K.
• Scale-up requires compliance with fertiliser contaminant limits, continuous pilot trials, and reliable LCAs.

## Graphical abstract

**Figure:**
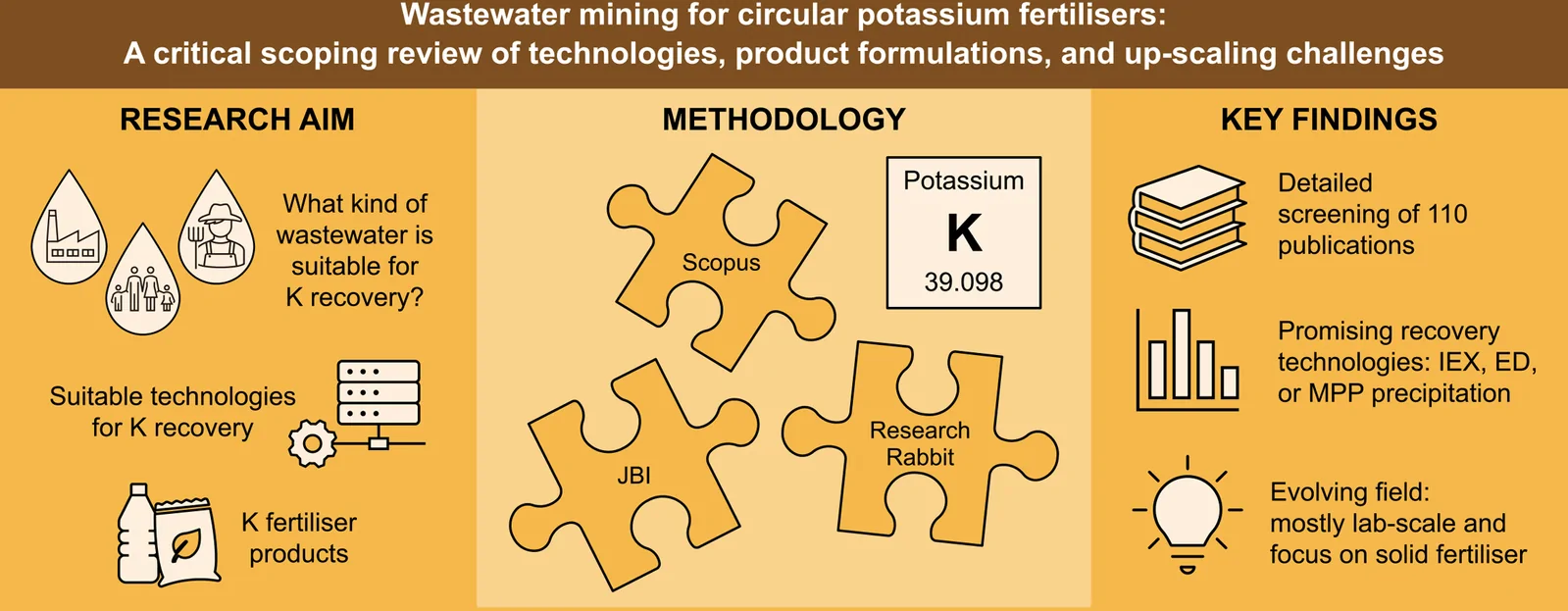

## 1 Introduction

Global agriculture faces the combined challenges of increasing food demand, excessive fertiliser use and the growing effects of climate change [1]. Improving nutrient-use efficiency while maintaining adequate nutrient supply is therefore essential for food security and sustainable agricultural production. Alongside phosphorus (P) and nitrogen (N), potassium (K) is an essential plant macronutrient with important roles in plant nutrition and public health [2 3], but produced predominantly from non-renewable sources. In plant nutrition, K supports photosynthesis, improves water use efficiency, influences carbohydrate and sugar formation and transport, and increases plant resistance [[2], [3], [4], [5]]. In humans, K deficiency can cause fatigue, muscle weakness, constipation, altered consciousness, or cardiac arrhythmia [2 [6]. The World Health Organisation therefore recommends a daily K intake of 3.5 g per day for adults [6].

Global potash production reached 42,914,224 t potassium oxide (K_2_O) in 2022, with an average application rate of 21.7 kg ha^−1^ of cropland [7]. Fertiliser K use was particularly high in North America (23.5 kg ha^−1^), South and Central America (36.8 kg ha^−1^), and Asia (28.3 kg ha^−1^) [7]. Although the estimated availability of geological K resources may suggest limited immediate pressure to replace conventional K fertilisers [8], economic and geopolitical factors can constrain their accessibility. Prices for muriate of potash (MOP) have increased since 2020, reaching an average of USD 880.1 t^−1^ in 2022, compared with a previous average of USD 353.3 t^−1^, and have not yet returned to pre-2020 levels [9 10].

Commercial K fertilisers are traditionally produced from mined mineral resources, predominantly as potassium chloride (KCl; MOP), usually mined from sylvinite ores, or potassium sulphate (K_2_SO_4_; sulphate of potash, SOP). K fertilisers contain approximately 14–61% K_2_O by weight (weight percentage, wt%), with MOP typically containing approximately 60 wt% K_2_O [11]. Although K is supplied to plants predominantly as K^+^, the K content of commercial fertilisers is conventionally expressed as the equivalent mass percentage of K_2_O [12]. In 2022, the largest shareholders and suppliers of potash were Canada (32%), China (15%), Russia (12%), Germany (10%), and Belarus (9%) [13]. Consequently, most fertiliser users depend heavily on a few global players. This dependence is particularly pronounced in the Southern Hemisphere, and K deficiencies in soils have also been reported in parts of Africa and Asia [12 14].

Potash mining can also exert substantial environmental pressures, especially on surrounding water bodies next to mining facilities [15]. Typical hazards are leaching from still unprotected stockpiles, which consist of residuals from mining with mostly sodium chloride (NaCl), increasing groundwater salinity due to leaching from injected salty wastewater stored in deeper soil layers such as plate dolomite, or discharge of saline wastewater directly into nearby water bodies such as the Werra River in Germany [[16], [17], [18], [19], [20], [21]]. High salinity in surface water bodies can alter biological processes and communities. In river ecosystems, it can increase cyanobacteria concentrations [[22], [23], [24], [25]]. For example, blooms of fish-toxic golden algae have been reported at chloride (Cl^−^) concentrations of 350 mg L^−1^ and can cause gill deformation in fish [26] 27]. In Germany, a K concentration limit of 20 mg L^−1^ (90th percentile) has been established for the Werra and Weser Rivers, both of which are affected by potash-mining activities, based on a five-class assessment system under the European Union Water Framework Directive [19]. Climate warming may further increase evaporation and river salinity, amplifying negative effects [28].

Consequently, alternative sources of K should be explored to reduce the environmental impacts of conventional K mining, for example by substituting part of the demand with K fertilisers recovered from wastewater. For instance, in Germany, a wastewater treatment plant (WWTP) serving 100,000 population equivalents (PE), with an average daily inflow load of 4.9 g PE^−1^ d^−1^ [29], could theoretically recover up to 490 kg K d^−1^ as fertiliser depending on the technology and its efficiency. To assess the potential of different wastewater types for K fertiliser production, this review provides an overview of where, to what extent, with which technological approaches, and in what form K could be recovered from wastewater.

However, knowledge of K recovery remains fragmented across wastewater streams, technologies, and potential products. A preliminary search of the Scopus database using the terms “potassium AND recovery AND wastewater” returned 1236 publications, including ten review articles substantially related to K recovery. Existing reviews have generally addressed specific technologies or K sources rather than K recovery across wastewater origins and treatment approaches. Yesigat et al. [30] and Kabdaşlı et al. [31], for example, reviewed potassium struvite (MPP), one route for K recovery from wastewater (see Section 3.4). Other K-related reviews have investigated K-rich crop residues as soil amendments [32 33], the development of alternative K resources [12 34], or the use of industrial de-dusting residues as a source of K fertiliser production [35]. Arienzo et al. [36] reviewed the use of K-containing wastewater for irrigation, but this review predates more recent regulatory developments on water-reuse quality and the monitoring of organic and inorganic pollutants. Broader reviews of resource recovery from wastewater are also well researched; however, they mainly focus on recovering N and P or on a single wastewater stream, such as urine [[37], [38], [39], [40]].

To address this gap, this scoping review maps available evidence and provides an overview of current K recovery methods in wastewater. All research studies were considered regardless of scale and technology readiness level. A scoping review was selected since research around K recovery is highly heterogeneous. Specifically, this review examines: (i) the current status and potential of wastewater-derived K recovery for fertiliser production; (ii) where and through which processes K is recovered within wastewater treatment systems; (iii) the recovery efficiencies and technological maturity of existing approaches; (iv) the scales at which these technologies have been demonstrated; (v) the fertiliser products generated from different wastewater streams and recovery processes; and (vi) the suitability of recovered solid and liquid fertilisers for different applications and relevant regulatory requirements.

## 2 Material and methods

### 2.1 Review framework and eligibility criteria

To ensure a holistic, systematic, and transparent literature selection process while minimising potential bias, this scoping review followed the Joanna Briggs Institute (JBI) methodology for scoping reviews (updated version, 2020) [41]. The review framework was defined according to the PCC (Population, Concept, and Context) principle:

⁃ Population: wastewater
⁃ Concept: potassium recovery
⁃ Context: reuse as fertiliser

Based on this framework, studies were considered eligible when they investigated wastewater as a source for K recovery and when the recovered products or processes were relevant to potential fertiliser applications. Table 1 summarises the detailed inclusion and exclusion criteria.

**Table 1:** Inclusion criteria regarding types of sources of evidence.

No.	Criteria	Explanation
1	Keywords: wastewater (all kinds, including synthetic wastewater), K recovery (all approaches), and reuse as fertiliser (all applications)	Defined according to the PCC framework (Population, Concept and Context).
2	Peer-reviewed research articles, review articles, and book chapters	K recovery from wastewater is an emerging research field and has not yet been widely implemented at wastewater treatment plants, where nutrient-recovery efforts have mainly focused on P and N.
3	Focus on quantitative data	To assess the current state of K-recovery technologies and enable comparison among studies.
4	Peer-reviewed publications from all years	To capture the development of K-recovery research over time.

### 2.2 Literature search and screening

We conducted literature searches in the Scopus database using the Boolean search terms “potassium AND recovery AND wastewater.” The term “reuse as fertiliser” from the PCC framework was not included in the search strategy to provide an overview of K recovery in general and to avoid excluding publications that do not mention further use of recovered K. No additional restrictions, such as document type, were changed or limited. We chose Scopus instead of Google Scholar because it offers better selectivity for the environmental and engineering sciences field [42]. Publications were initially screened based on titles and abstracts, followed by full-text assessment according to the predefined exclusion criteria:

⁃ Potassium, potash, or K^+^ (mg L^−1^) was not found in the publication.
⁃ K^+^ was mentioned only once as a macronutrient, and the publication's main focus was unrelated.
⁃ The publication did not use wastewater, including sludge, manure, or synthetic wastewater, as a recovery source.
⁃ No K-recovery process was investigated.
⁃ The publication was not written in English.
⁃ The publication was a duplicate.

We recorded the selected publications in Microsoft Excel (Microsoft 365) during the review process, including authors, DOI, publication year, and preliminary findings. These data supported subsequent analyses, including evaluating research development trends (Section 3.1).

Furthermore, the pre-selected publications were expanded using Research Rabbit, an artificial intelligence (AI)-assisted literature discovery tool that explores citation networks to identify related publications [43]. Research Rabbit was used to identify relevant earlier and later studies based on the selected publications. This additional search approach was applied to reduce potential database bias, as the primary database used in this review (Scopus) may not capture all relevant publications [42]. Unlike a conventional database search, Research Rabbit relies on citation connections and therefore may overlook studies with limited citation links. Thus, Research Rabbit was used as a complementary tool rather than a substitute for the systematic database search. Publications identified through Research Rabbit were screened using the same exclusion criteria described above.

### 2.3 Data extraction and categorisation

The remaining publications were imported into the literature software Citavi (version 6.18.0.0) for further organisation and data extraction. Citavi was used to organise relevant information and link extracted knowledge (citations) to the corresponding research questions. Studies were categorised according to the following characteristics:

⁃ research step;
⁃ publication type;
⁃ wastewater origin;
⁃ K-recovery technology;
⁃ technology readiness level;
⁃ recovered K product.

The detailed classification scheme and subcategories identified from the literature are provided in Supplementary Text S1.

### 2.4 Data synthesis

Following JBI recommendations, data synthesis in this scoping review was descriptive rather than based on quantitative meta-analysis [41]. We summarised the extracted information using figures, tables, and descriptive analyses to identify trends in wastewater sources, recovery technologies, technological maturity, and fertiliser applications. A summary of the review characteristics and extracted information is provided in Supplementary Fig. S1.

## 3 Results and discussion

### 3.1 Selection of publications

A Scopus search conducted on 23 April 2025 using the terms “potassium AND recovery AND wastewater” identified 336 publications without additional restrictions. After removing duplicates and applying the predefined screening criteria, we retained 52 publications for further assessment. To complement the database search, we analysed the pre-selected publications using Research Rabbit, which identified additional related studies through citation networks. After screening 225 records identified through earlier and later citation links, we included 63 additional publications. In total, we assessed 115 publications; five could not be accessed and were therefore excluded. The final dataset comprised 110 publications (Fig. 1).

**Fig. 1:**
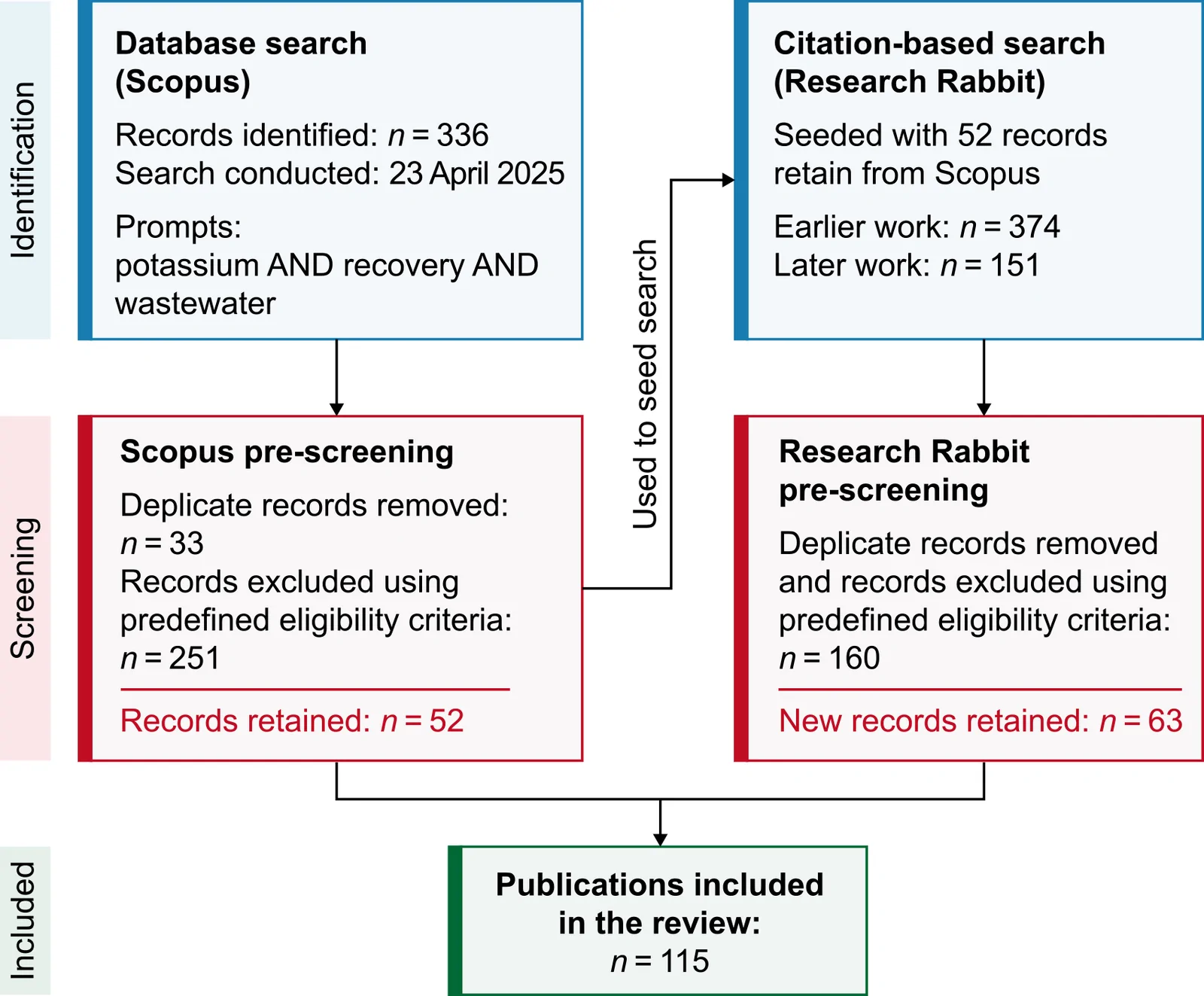
Identification and selection of publications included in the review. Flow diagram showing the Scopus database search, citation-based exploration using Research Rabbit, removal of duplicate and ineligible records, and final inclusion of 115 publications.

To contextualise the development of K recovery research, we conducted a comparable Scopus search using “phosphorus AND recovery AND wastewater” on the same date, yielding 1904 publications. Thus, the number of publications related to P recovery was approximately six times higher than that related to K recovery, indicating that wastewater-based K recovery remains a comparatively less explored research area.

### 3.2 Types of publication and technology level

The number of publications related to K recovery in wastewater is visualised as a timeline, showing a total of 110 publications and the number of reviews or books (Fig. 2a), and the kind of technology level investigated (Fig. 2b). Fernandez et al. [44] conducted a study at the beginning of the nineties regarding co-ion behaviour of cationic (including K) ion exchange at high concentration in industrial wastewater. Kirchmann and Pettersson [45] investigated human urine regarding chemical composition, including K, and its usability as a fertiliser without advanced technical treatment. Since 2016, publications on this review topic have increased, peaking in 2023 with 15 publications. The total number of publications for 2025 represents the period up to 23 April 2025.

**Fig. 2:**
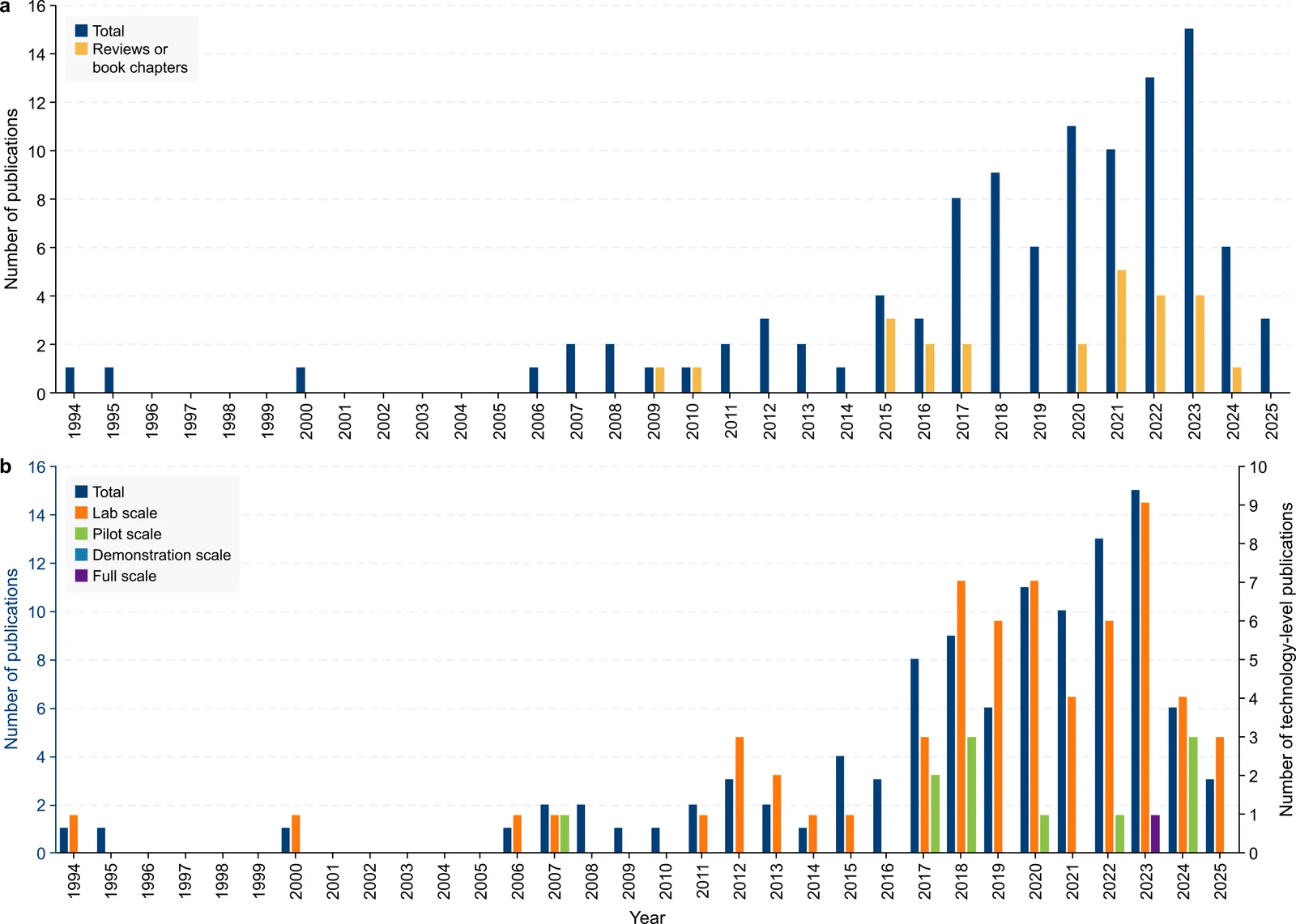
Temporal trends in publications on potassium recovery from wastewater. a, Annual numbers of all publications and publications classified as reviews or book chapters between 1994 and 23 April 2025. Reviews and book chapters are included in the total number of publications. **b**, Annual number of publications and distribution of studies among lab-, pilot-, demonstration-, and full-scale applications. The left *y*-axis indicates the total number of publications, and the right *y*-axis indicates the number of publications at each technology scale. Data for 2025 include publications available up to 23 April 2025.

In general, 109 peer-reviewed research articles have been found, including review articles (20 publications). As reported in Section 1, review articles cover resource recovery from wastewater in general, but not specifically K recovery from various wastewater streams. Furthermore, one doctoral thesis by Johansson [46] on using autotrophic N removal for K and P removal from municipal wastewater was found; however, it focuses on K removal, not K recovery. One book chapter by Hidayat and Harada [47] is among the identified publications on K-struvite.

The experimental scales reported in the publications are shown in the timeline (Fig. 2b). Most articles investigate lab-scale (61 articles) or pilot-scale (11 articles), as defined by the authors of the respective study, which shows that research on K recovery is still evolving. Furthermore, the classification of whether an experiment is lab-scale or pilot-scale is described very vaguely. The publications provide no definitions, and there is no clear, systematic boundary, as attempted with the method technology readiness levels [48]. Specific requirements and definitions for each scale would be helpful for a better comparison of research status. Parameters such as flow rates, loads, plant sizes, or specific wastewater requirements (e.g., synthetic wastewater allowed with defined requirements) for each scale would help distinguish and compare the scales. Some of the pilot scales considered were carried out with synthetic wastewater [49]. No demo-scale was found. However, one large-scale application is defined in the publications. The study investigates a combination of pre-ammonium recovery through a steam distillation column and post-K recovery through struvite precipitation (Section 3.4) from a one m^3^ source-separated urine storage tank collected from a public restroom [50 51]. Given the defined plant sizes for each scale, this study would likely not be considered a full-scale plant. Other publications either did not mention a technology level or were review papers. A trend across publications, e.g., fundamental research at the beginning due to process behaviour, is not apparent. Overall, the series has shown that K recovery from wastewater as an emerging resource has gained attention in the last couple of years.

### 3.3 Types of wastewater

Wastewater streams vary considerably in composition, flow characteristics and nutrient-recovery potential. Concentrated streams, such as source-separated urine and digestate, differ fundamentally from dilute municipal wastewater streams, creating distinct opportunities and challenges for K recovery. Detailed descriptions of wastewater treatment systems and resource-oriented sanitation approaches are available elsewhere [[52], [53], [54]].

Most research on K recovery has focused on urine, including synthetic urine (48 publications, including ten review papers or book chapters). However, six research articles did not specify a K recovery technology (Section 3.4) and are not included further. Livestock wastewater, including manure and slurry (11 publications), has also been investigated more frequently, while other wastewater streams were examined only sporadically (Fig. 3).

**Fig. 3:**
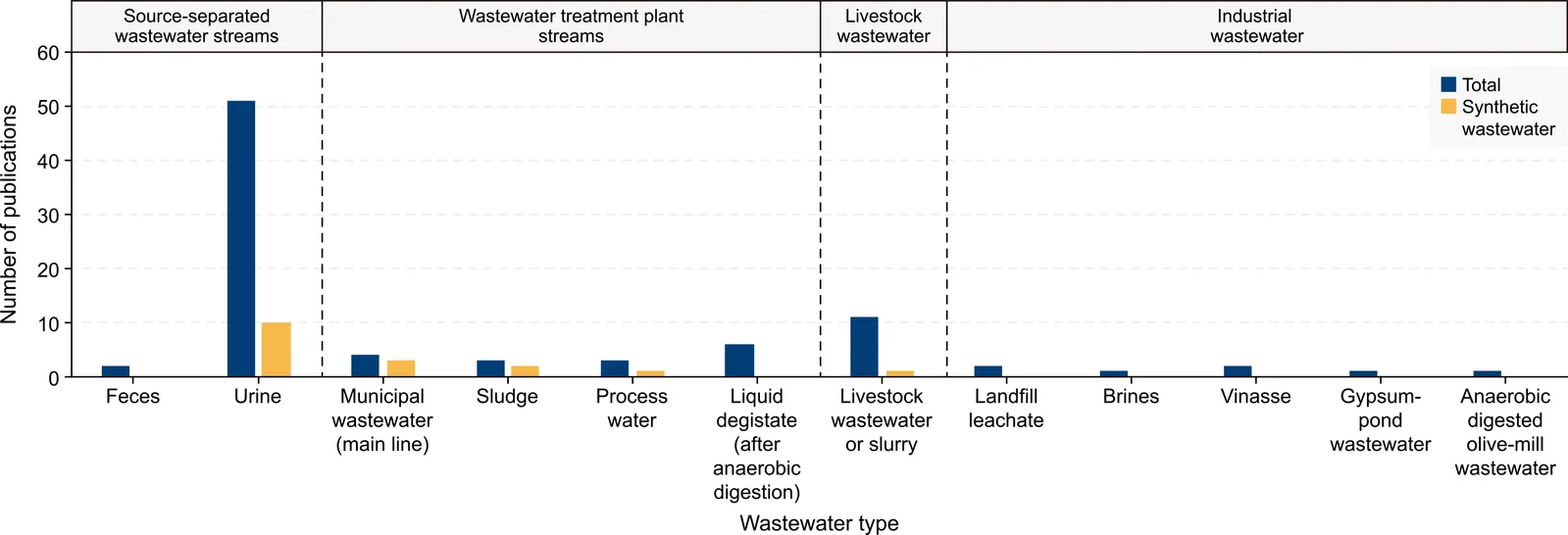
Wastewater types investigated for potassium recovery. Number of publications examining different real and synthetic wastewaters. Wastewater is grouped into four source categories: resource-oriented wastewater side streams, streams within wastewater treatment plants, livestock-derived agricultural wastewaters, and industrial wastewaters. Blue bars indicate the total number of publications, and yellow bars indicate studies using synthetic wastewater.

Most research is conducted in wastewater streams such as urine and livestock wastewater due to high K concentrations (mostly above 1000 mg L^−1^; Table 2), but their flow rates are substantially lower than those of the main stream of municipal WWTP.

**Table 2:** Total K concentrations in different wastewater types.

Type of wastewater	Wastewater subcategory	Range (mg L^−1^)	Mean ± SD (mg L^−1^)	Reference
Urine	Fresh	-	1682	[55]
Urine	Fresh	-	1720	[56]
Urine	Fresh	-	1470	[57] 58]
Urine	Hydrolysed	-	1773	[55]
Urine	Hydrolysed	-	1336	[59]
Urine	Stored	-	1081	[60]
Urine	Stored	-	1410 ± 320	[61]
Urine	Stored	900–1100	-	[45]
Urine	Synthetic	-	267 ± 59	[62]
Livestock wastewater	Swine manure	-	757 ± 338	[50]
Livestock wastewater	Pig manure	-	1584	[63]
Livestock wastewater	Pig manure	-	1600 ± 300	[64]
Livestock wastewater	Cattle manure∗	-	1000	[65]
Livestock wastewater	Cattle manure∗ after membrane treatment	-	1535	[66]
Livestock wastewater	Manure∗	-	892 ± 103	[67]
Livestock wastewater	Swine wastewater	-	384 ± 13	[68]
Industrial wastewater	Landfill leachate	1105–2141	1933 ± 276	[69]
Industrial wastewater	Olive mill wastewater∗ after centrifugation and filtration	-	1969 ± 10	[70]
Industrial wastewater	Semiconductor wastewater	9–49	-	[71]
Industrial wastewater	Vinasse	-	1390 ± 8	[72]
Industrial wastewater	Vinasse after ultrafiltration	-	1008 ± 36	[73]
WWTP wastewater streams	Primary settled wastewater	-	25 ± 0.1	[74]
WWTP wastewater streams	Centrate	-	251 ± 2	[75]
WWTP wastewater streams	Liquid digestate∗	2185–2440	-	[76]
WWTP wastewater streams	Liquid digestate∗	1494–2337	1981	[77]
WWTP wastewater streams	Liquid digestate∗	-	636 ± 20	[78]
WWTP wastewater streams	Synthetic sewage sludge effluent	-	113	[79]

Note: Total K concentrations are reported as ranges or mean values (rounded), as presented in the original studies. Standard deviations are shown where available. -, not reported.∗Wastewater after anaerobic digestion. WWTP, wastewater treatment plant.

These characteristics make them attractive targets for K recovery. Furthermore, urine does not interfere with industrial wastewater and contains minimal solid compounds. Livestock wastewater exhibited a wide range of characteristics, reflecting variation among animal sources (Fig. 3). Additionally, livestock wastewater or manure includes both urine and faeces and therefore solid compounds [80]. Nevertheless, these streams are generally more clearly defined than municipal WWTP influent, which can contain a mixture of domestic and industrial wastewater.

K concentrations reported for different wastewater streams in the publications are summarised in Table 2. Because possible co-ions (e.g., N or P) for recovery or counter-ions (e.g., Sodium (Na)) may inhibit K recovery, their potential effects are summarised in Supplementary Table S1.

Regarding industrial wastewater, olive mill wastewater after anaerobic digestion and landfill leachate have the highest potential regarding K recovery due to high concentrations (above 1500 mg L^−1^). In general, a high standard deviation reported by Magrí et al. [64] shows high variation and suggests that K recovery technologies must be robust and flexible to fluctuations. However, many experiments used synthetic wastewater, which makes statements about upscaling at this point difficult, as important matrix effects and interference with counter ions are not taken into account yet (Fig. 3).

Regarding municipal wastewater, only four publications have studied the main line of a WWTP, including three that focus on synthetic wastewater. Measured concentrations in Table 2 vary widely across WWTP stages. High K concentrations (2185–2440 mg L^−1^) have been observed in liquid digestate, although the associated flow volume is relatively low [76]. In contrast, the effluent from primary sedimentation contains lower K concentrations (average 25 mg L^−1^) but represents a much larger water flux [74]. In WWTPs with enhanced biological P removal (EBPR), K can be incorporated partly into the biomass [81]. Johansson [46] mapped the nutrient flow over a WWTP with EBPR, showing that 80% of incoming K leaves again through effluent. Approximately 20% K are incorporated into biomass and can be released during digestion. Hofmann et al. [82] investigated nutrient recovery as liquid fertiliser from WWTPs for hydroponic cultivation. In this study, a literature-based model of a municipal WWTP, including chemical P removal and anaerobic sludge stabilisation, was set up, showing that, especially in the liquid mainstream, K currently flushes mainly through the WWTP [82].

For substances that might disrupt recovery and/or reduce fertiliser quality, K recovery at WWTPs could be advantageous in the effluent, producing liquid fertiliser or even single-nutrient fertiliser, if technologies can handle high flux and fluctuations. Using urine as a part-stream to produce K fertiliser is another approach if the aim is to produce a compound fertiliser. Urine is unsuitable as a single-nutrient fertiliser because of its high concentrations of N and P (Section 3.5).

Most research on urine also shows high concentrations of N and P, and much of it has been carried out without addressing K. Moreover, P is listed as a critical raw material, prompting research regarding resource recovery from wastewater for years [83].

In general, more research, especially with real wastewater, is needed to design technologies for K recovery robust due to fluctuations in wastewater composition.

### 3.4 Types of recovery technology

The selected literature covered a wide range of K-recovery technologies, with their respective frequencies of application (Fig. 4). In total, 94 identified publications investigated 11 different technologies. Wet-chemical processes, such as K-struvite precipitation (40 publications) and ion exchange (IEX) (15 publications), have been analysed most frequently. Electrochemical processes, such as electrodialysis (ED), have also been used in 17 publications. Physical processes such as membrane separation (e.g., forward osmosis (FO), reverse osmosis (RO), or membrane distillation (MD)) have also been used. Novel technologies such as hydrothermal liquefaction (HTL), hybrid capacitive deionisation (HCDI), and microbial fuel cells (MFCs) were rarely considered. Detailed information on individual studies is provided in Supplementary Table S2.

**Fig. 4:**
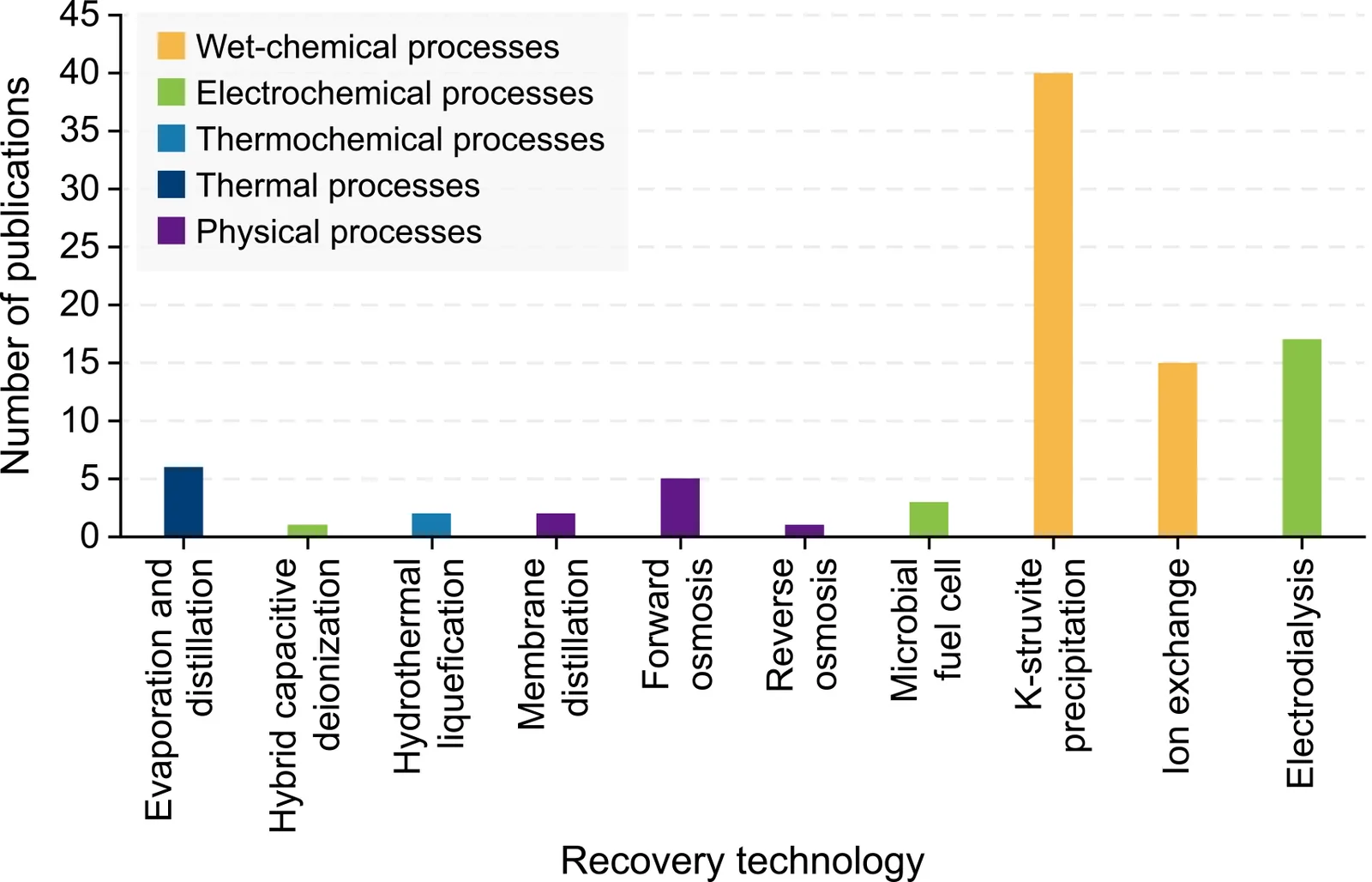
Technologies investigated for potassium recovery from wastewater. Number of publications examining recovery technologies classified as wet-chemical, electrochemical, thermochemical, thermal, and physical processes. Technologies are ranked according to the number of publications within each category.

K-struvite precipitation is the most commonly investigated approach (Fig. 4), particularly for wastewater with high concentrations of K and P, such as urine, livestock manure, and anaerobic digestion process water. The process involves adding magnesium (Mg) to K- and P-rich wastewater streams, leading to the crystallisation of Mg, K, and P (K-struvite; MPP; KMgPO_4_·6H_2_O) [84]. Counter ions, mostly ammonium (NH_4_^+^) and Na, lead to other struvite products, e.g., magnesium–ammonium–phosphate (MAP) or Na-struvite. Therefore, controlled precipitation needs favourable molar ratios. Previous studies have reported substantially different Mg:K ratios depending on the wastewater matrix and experimental conditions. For example, Bennett et al. [85] investigated synthetic wastewater and used a molar ratio of Mg:K:P of 3:50:1 to prevent other precipitation products, such as MAP. Another lab-scale study with real source-separated urine (comparing different magnesium sources) reported a K recovery of 70% with a molar ratio of Mg:K:P of 4:1:1.6, while inhibiting ammonia precipitation by adjusting pH to 11.5 with NaOH [86]. Furthermore, NH_4_^+^ removal beforehand leads to better molar ratios and a higher proportion of K-struvite [46].

Despite extensive investigation, no standardised molar ratio has been established for K-struvite precipitation, which varies by Mg:K, K:P, or Mg:K:P (Supplementary Table S2). For a product like MPP, which needs all three elements, a molar ratio that considers all three macronutrients should be meaningful. One lab-scale study with synthetic urine shows very good results [87] in the absence of NH_4_; however, scaling up and switching to real wastewater is more complicated because K and NH_4_ compete. Finding the right operating conditions seems highly dependent on the type of wastewater (including a complex matrix considering counterions) and the reactor type used. The pilot-scale investigation of Magrí et al. [64] with pig slurry shows large fluctuations in K recovery (15–51%) due to different Mg:K:P molar ratios, with K as a limiting factor and a risk of interference from free calcium. Moreover, Wilsenach et al. [84] investigated challenging settling characteristics of MPP at higher mixing speeds in a fluidised-bed reactor due to precipitant resuspension.

IEX is another wet-chemical process used for K recovery from wastewater. In general, ion exchange uses an insoluble solid matrix (adsorbent) containing mobile ions due to functional groups. Those ions can interact with other surrounding ions (depending on the affinity) through adsorption (e.g., chemisorption or physisorption processes) and desorption processes at the matrix [88]. Adsorbents can be either geogenic (e.g., clinoptilolite or chabazite) or industrial synthetic resins [70 89 90]. Wu et al. [91] tested different adsorbents (biochar, clinoptilolite, artificial zeolite, and chabazite) under lab-scale conditions, with the highest K recovery (91.2%) for Na-functionalized chabazite. For geogenic adsorbents and biochar, long-term stability should also be considered because of the potential for material leaching [58 91]. For good adsorption, the amount of counterions and the effective adsorption of K depend on wastewater characteristics and operating conditions, such as pH, temperature, adsorbent selectivity, cross-linking, bed volume (contact time), and loading [92]. Typical counter ions for K recovery with IEX, particularly Na and NH_4_^+^, are the same as for K-struvite precipitation due to their hydrated radii and diffusion coefficients [93]. K is more likely to be investigated as a counter ion [94]. No pre-treatment steps for K recovery using IEX to remove or minimise the effect of counter ions have been studied so far. As a combined process, Casadellà et al. [93] use in their study a clinoptilolite-based mixed-matrix membrane (MMM: 30 wt% polymer and 70 wt% clinoptilolite) in diluted urine as a combination of wet-chemical and physical processes for selective recovery of K and NH_4_^+^ and developed a desorption step at 60 °C, resulting in a K recovery of 60% (one-cycle proof of concept). Ciceri et al. [12] advise IEX and ion-selective membranes (like MMM) as a promising development to concentrate and recover K from wastewater. Most IEX research has been done at lab scale (Supplementary Table S2); most articles focus on adsorption processes, but little research has examined desorption aimed at recovering K in a soluble phase or the effect of counterions in both adsorption and desorption. In general, long-term tests on adsorbent stability and K selectivity and efficiency have not been done yet, which would be essential to investigate the effect of changing ion matrices or resins (e.g., through regenerants).

Membrane-based processes have also been investigated for K recovery, primarily as filtration and concentration steps. Relevant overviews of membrane technologies are provided by Jafarinejad [95] and Obotey Ezugbe and Rathilal [96]. Zhang et al. [97] investigated a lab-scale FO for source-separated urine as a dewatering step for mining nutrients, including N, K, and P. Reverse osmosis (RO) has likewise been applied to liquid digestate after anaerobic digestion [77], while FO has been used to concentrate nutrients from urine for subsequent algal cultivation [98]. Although these concentration steps contribute to a high recovery rate of 79–97% [97], micropollutants and heavy metals might also concentrate and should be considered. Further operational problems include fouling and scaling, and FO has a lower affinity for fouling due to lower operating pressures [95].

Tran et al. [78] combined ED with FO in a lab-scale study of liquid digestate after anaerobic digestion, achieving 86% K recovery at a concentration factor of 3.6.

ED is the second most frequently investigated technology for K recovery (Fig. 4). ED is an electrochemical process employing membranes with an electrical potential difference. Therefore, ions, based on their charge (cations or anions), diffuse in different chambers [99]. Process performance depends on membrane configuration, the number of compartments and the ionic composition of the wastewater. Li et al. [67] used a synchronous cationic- and anion-selective ED for digestive slurry and reached 70% K recovery at 7 V. Other studies investigated ED for K-struvite crystallisation in vinasse with up to 68% K recovery [72 73]. Regarding product quality (e.g., fertiliser), it would also be helpful to know the molar ratio of initial concentrations (Section 3.5). Overall, ED can achieve high recovery depending on the membrane type.

Other technologies include evaporation, distillation, HTL, HCDI, and MFC (Fig. 4). Thermic processes, such as evaporation and distillation, have been used solely for K recovery in urine. Simha et al. [100] studied alkaline dehydration of stabilised urine using wood ash or biochar as an alkaline medium and reached a lab-scale K recovery of 100%. Conti et al. [101] studied thermochemical processes like HTL to produce biochar of willow, which was irrigated by synthetic secondary effluent. In the process, K is recovered (95%) as a byproduct in the aqueous phase of the HTL [101]. Electrochemical concepts, such as HCDI and MFC, remain novel wastewater technologies. HCDI has been used in synthetic wastewater, achieving 36% K removal at 0.1 V at lab scale [102]. MFCs have been used in swine wastewater, with K recovery (as energy use) of 44–49% when comparing flooded and tidal operation modes [103].

A comparative overview of the K recovery technologies identified in this review, including wastewater type, technology stage, influent K concentration, K removal, K recovery efficiency, product form, and investigation type is provided in Table 3.

**Table 3:** Overview of K-recovery technologies, including technology stage, wastewater type, influent K concentration, K removal, K recovery efficiency, product form, and experimental duration.

K recovery technology	IEX	K-struvite precipitation	ED	MFC	HCDI	HTL	Evaporation or distillation	RO	FO
Number of publications									
All publications	9	23	7	1	1	1	5	1	5
With synthetic wastewater	2	13	-	-	1	1	-	-	1
Technology stage reported in the publications									
Lab scale	6	18	5	1	1	1	5	-	4
Pilot scale	1	2	2	-	-	-	-	1	1
Demonstration scale	-	-	-	-	-	-	-	-	-
Full scale	-	1	-	-	-	-	-	-	-
Not reported	2	2	-	-	-	-	-	-	-
Wastewater type reported in the publications									
WWTP mainstream	1 (1 syn.)	3 (3 syn.)	-	-	1 (1 syn.)	1 (1 syn.)	-	-	1 (1 syn.)
WWTP sludge line	1 (1 a.d.)	1 (1 a.d.)	2 (1 a.d.)	-	-	-	-	1 (1 a.d.)	1 (1 a.d.)
Industrial wastewater	2 (1 a.d.)	-	2	-	-	-	-	-	-
Livestock wastewater	-	6 (2 a.d., 1 syn.)	1	1	-	-	-	-	1
Source-separated urine	3 (1 syn.)	12 (9 syn.)	2	-	-	-	3	-	2
Brines	1	-	-	-	-	-	-	-	-
Influent K concentration range (mg L^−1^) reported in literature	4–3382	9–2610	165–3400	400–2600	-	891–946	1023–1410	25–1981	25–2720
K removal from wastewater (%) reported in literature	37–78	-	-	-	36	-	-	90	-
K-recovery efficiency (%) reported in literature	43–100	15–98	13–95	44–49	-	95	6–100	-	79–97
Recovered product form	Liquid	Solid	Solid or liquid	Liquid	Only removal	Solid	Solid	Liquid	Liquid
Experimental mode	Single runs (9 pub.), continuous test (1 pub.)	Single runs (19 pub.), continuous test (5 pub.)	Single runs (5 pub.), continuous test (2 pub.)	Continuous test (1 pub.)	Single runs (1 pub.)	Single runs (1 pub.)	Single runs (4 pub.)	Continuous test (1 pub.)	Single runs (3 pub.), continuous test 3 pub.)
Duration of continuous tests	4-column cycles	2–4 h; 3 h; 10 h; 8 h; 70 days	40 h, 3 months	63 days	-	-	-	3 years	70–100 h; >20 h; 48 h

Note: The table summarises reported K-recovery technologies according to the information available in the reviewed publications. The values represent the ranges reported in the literature and are not intended for statistical comparison. -, not reported. #a.d., number of publications using wastewater after anaerobic digestion; #syn., number of publications using synthetic wastewater; #pub., number of publications. The information on influent K concentrations, K removal efficiencies, and K-recovery efficiencies are reported in the literature and do not represent statistical distributions. IEX, ion exchange; ED, electrodialysis; MFC, microbial fuel cell; HCDI, hybrid capacitive deionisation; HTL, hydrothermal liquefaction; RO, reverse osmosis; FO, forward osmosis.

Most research remains lab-scale and uses synthetic wastewater. It also shows the wide range of wastewater streams, including both synthetic and real wastewater, and the concentrations reported in the publications. Detailed statistics on influent K concentrations and competing ions are provided in Table 2 and Supplementary Table S1–2. Future research with synthetic solutions should also consider counterions. The summary of influent concentrations in Table 3 illustrates the range covered and shows that each process has its own conditions depending on wastewater type. For IEX, we investigated the widest K influent concentration range (4–3382 mg L^−1^). Most K recovery technologies have very little information on K removal from wastewater. For IEX, K removal ranges from 37% to 78%, depending on wastewater type and interfering counterions. In studies of HCDI and RO, researchers considered only removal; they did not consider recovery. Regarding K recovery efficiency, all technologies show a broad range of efficiencies, ranging from 13% to 95% for ED and 15% to 98% for K-struvite precipitation. These ranges suggest potential for further development of K recovery technologies, but more research is needed. The potential forms of the K recovery products are listed in Table 3. IEX, MFC, RO and FO primarily yield liquid products, whereas K-struvite precipitation, HTL and evaporation produce solid products. Both solid and liquid products could be produced from ED. The overview of experimental methods used in the publications also shows that research into K recovery from wastewater is still in its infancy. Most studies were based on single-run experiments, with few long-term trials; extended studies lasting several months have mainly been reported for membrane-based processes and K-struvite precipitation.

Potential challenges for full-scale implementation, with the system boundary defined by the inlet and outlet of each technology, are displayed in Table 4. Possible challenges include scaling, fouling, chemical demand (due to additional process chemicals, regenerants, antiscalants, and cleaning agents), high energy demand, operational complexity, process stability, and sensitivity to influent composition (e.g., counter-ion interference). Fouling and scaling are major concerns for membrane-based technologies, while scaling may also occur on equipment (e.g., stirrers) for MPP precipitation. Most K-recovery technologies require chemical inputs, although the extent of this demand varies by process. Sensitivity to influent concentrations (due to counter ions) depends heavily on the wastewater type and the desired product (Section 3.5). High energy demand may constrain ED, HCDI, HTL, evaporation or distillation, and RO, although greater use of renewable energy could reduce the associated environmental burden.

**Table 4:** Potential challenges associated with full-scale implementation of K-recovery technologies.

K recovery technology	IEX	K-Struvite precipitation	ED	MFC	HCDI	HTL	Evaporation or distillation	RO	FO
Potential challenges for full-scale implementation									
Fouling	-	-	+	+	○	-	-	+	+
Scaling	-	+^g^^,^^i^	+	+	+	-	+	+	+
Chemical demand (additional chemicals, regenerants, antiscalants, and cleaning agents)	+^k^	+^b^	+^b^	○	+	○^a^	○^a^	+^b^^,^^j^	+^b^^,^^j^
High energy demand	○^e^	○^f^	+	-	+	+	+	+	-
Operational complexity	○^d^	+^h^	○	○	○	○	○	○	○
Process stability	○	+^c^	+^c^	○	○	○	+	+^c^	+^c^
Sensitivity to influent composition and competing ions	+^d^	+	+^c^	+	○	○	-	+^c^	+^c^
Authors' assessment									
Scale-up feasibility	×	×	×	×	×	×	×	✓	✓

Note: The system boundary includes the inlet and outlet of each recovery technology. The assessment represents the authors' qualitative evaluation of scale-up feasibility based on the current research status. IEX, ion exchange; ED, electrodialysis; MFC, microbial fuel cell; HCDI, hybrid capacitive deionisation; HTL, hydrothermal liquefaction; RO, reverse osmosis; FO, forward osmosis. Symbols indicate: -, unlikely to represent a major challenge; ○, uncertain whether it represents a challenge; +, potential challenge; ×, insufficient evidence to assess scale-up feasibility; ✓, scale-up feasibility has been demonstrated in related applications.

Footnotes:

a pH adjustment;

b Cleaning requirements;

c Fouling and scaling-related effects;

d Depends on the target K-recovery product;

e Backwashing and pump-assisted feeding requirements;

f Mixing requirements;

g Equipment scaling;

h Strongly dependent on wastewater type;

i Precipitant requirements;

j Antiscalant requirements;

k Regenerant requirements.

An initial assessment of the fundamental scalability of these processes is provided in Table 4, which have so far been predominantly based on laboratory experiments. This assessment is intended only to provide an initial indication of the effort required to implement these technologies at scale and the challenges to consider. Technologies such as RO or FO have already been implemented at scale in others, though partly similar, contexts, so scaling up seems fundamentally feasible. Other technologies, such as IEX or ED, show through their current research that proof-of-concept is a K recovery technology. An assessment of whether scaling up those technologies is feasible is not possible at this level of knowledge.

No technology combinations has been investigated yet (see Table 4). Although integrated process trains may increase operational complexity, they can also create useful synergies. For example, WWTP effluents combine high flow rates with relatively low K concentrations, suggesting that dilute streams may require a multi-step process configuration that first increases K concentrations to facilitate the second recovery step. A membrane-based concentration step, for instance, could improve the feasibility of a subsequent IEX process for K recovery. Challenges associated with the membrane stage, such as scaling and fouling, could be resolved by incorporating an upstream softening stage or antiscalants. As the field develops, the assessments summarised in Table 3, Table 4 should be updated to reflect advances in process integration and scale-up.

This review did not consider investment or operating costs, as a reliable assessment based primarily on laboratory tests (Table 3) is not possible. K recovery from wastewater remains a niche area and all technologies for K recovery are still in an early stage. The primary aim of developing new processes in the wastewater sector should be to protect the environment and to develop new, circular economy-oriented processes.

### 3.5 Type of K fertiliser

Solid fertilizers were reported in 52 publications, comprising K-struvite (*n* = 41), biochar (*n* = 6), and products whose composition was not defined (*n* = 5). The focus on K-Struvite reflects the current focus on slow-release fertilisers. As already indicated in Section 3.4 K adsorbtion by biochar is relatively limited.

Liquid fertilizers were reported in ten publications, such as KCL or KOH, although their compositions were not specified. Furthermore, Hofmann et al. [82] identified a research gap in using liquid K or N-P-K fertiliser from wastewater for hydroponics. Moreover, Ciceri et al. [12] suggest algae, such as kelp, as an additional type of K fertiliser.

Identified K fertiliser products in the reviewed literature are summarised in Table 5 according to product type, K content, wastewater origin, and K recovery technology. However, studies report K content inconsistently, using units such as weight percentage, molar percentage, and mg L^−1^, which limits direct comparison among products. Reporting K content on a consistent mass basis, for example as weight percentage or K_2_O equivalent, would facilitate comparison with conventional K fertilisers, which typically contain 14–61 wt% K_2_O (Section 1). Only four studies reported K content on a weight-percentage basis. For example, Harada et al. [104] produced a fertiliser with 18.4% K_2_O using synthetic urine. However, these results were gained under optimal conditions without environmental impact. Nine studies did not mention the proportion of K, while the remaining studies used other units.

**Table 5:** K fertiliser products identified in the literature, including product type, reported K content, wastewater source and recovery technology.

Fertiliser type	Fertiliser description in publications	Proportion of K in fertiliser	Type of wastewater	K recovery technology	Reference
Liquid	KCl	n.s.	Synthetic urine	IEX	[93]
Liquid	KOH	1008.5 ± 6 mg L^−1^ in adsorption chamber. Mixture of K^+^, H^+^ NH_4_^+^ and SO_4_^2−^	Urine	ED	[105]
Liquid	Cathodic solution with K, N, Na, Mg, and P	225–150 mg L^−1^ in cathodic solution	Swine wastewater	MFC	[103]
Liquid	K_2_O in N–P–K mixed solution through acidic pathway	Nutrient-rich liquid organic fertiliser containing 1.3% N, 1.3% P_2_O_5_, and 1.5% K_2_O; 68.7 g of fertiliser per kg of treated slurry	Pig slurry	FO	[106]
Liquid	N–P–K mixed solution	Final ED concentration: 2658.3 ± 30.5 mg L^−1^; final FO draw concentration: 554.7 ± 19.7 mg L^−1^	Liquid digestate after anaerobic digestion	ED–FO	[78]
Liquid	NH_4_^+^ and K solution in concentration stream	4571.4 mg L^−1^	Digestive slurry	ED	[67]
Liquid	Aqueous HTL by-product	13,339 ± 2229 mg L^−1^	Secondary effluent	HTL	[101]
Solid	Biochar	Elemental composition of end-products reported on a dry matter basis (wt%). At 50 °C, ash and bran ash were 7.7% and 6.9%, respectively; at 60 °C, they were 8.4% and 6.1%, respectively.	Urine	Alkaline dehydration on biochar	[107]
Solid	Biochar	8.5 g K per 188.3 g product; ash: 1.4 g K per 172 g product	Urine	Alkaline dehydration on biochar	[100]
Solid	MPP	n.s.	Pig slurry	Struvite precipitation	[63]
Solid	MPP	K-struvite purity approximately 60% at Mg:K:P 1:1:1 with a pH of 11	Synthetic urine	Struvite precipitation	[108]
Solid	MPP	n.s.	Synthetic Urine	Struvite precipitation	[62]
Solid	MPP	36.0% PO_4_-P, 18.4% K, and 45.6% Mg	Livestock wastewater	Struvite precipitation	[104]
Solid	MPP	n.s.	Synthetic livestock wastewater	Struvite precipitation	[47]
Solid	MPP	55 mg L^−1^ in precipitates at Mg:K 1.5:1	Urine	Struvite precipitation	[86]
Solid	MPP	n.s.	Urine	Struvite precipitation	[60]
Solid	MPP	10–12% in K-struvite	Urine	Struvite precipitation	[51]
Solid	MPP	n.s.	Urine	Struvite precipitation	[109]
Solid	MPP	n.s.	Urine	Struvite precipitation	[110]
Solid	MPP	5.0% K	Pig slurry	Struvite precipitation	[64]
Solid	MPP	n.s.	Synthetic sewage sludge effluent	Struvite precipitation	[79]
Solid	MPP	n.s.	Liquid digestate after anaerobic digestion	ED, MF, UF, and RO	[77]
Solid	MPP	15.7 mol% K	Livestock wastewater after secondary treatment	Struvite precipitation	[111]
Solid	MPP	9.0–9.8 wt% K	Urine	Struvite precipitation	[112]
Solid	MPP	3.2 kg K-struvite m^−3^	Vinasse	Struvite precipitation	[73]
Solid	MPP	8.4 wt% K	Swine wastewater	Struvite precipitation	[68]
Solid	MPP	Purity > 67.7 ± 3%; particle size: 1 mm	Swine wastewater	Struvite precipitation	[113]
Solid	MPP	n.s.	Synthetic urine	Struvite precipitation	[87]
Solid	MPP	n.s.	Synthetic urine	Struvite precipitation	[114]
Solid	MPP	n.s.	Synthetic urine	Struvite precipitation	[115]
Solid	MPP	n.s.	Source-separated urine	Struvite precipitation	[49]

Note: n.s., not specified; MPP, magnesium potassium phosphate (K-struvite; KMgPO_4_·6H_2_O). K contents are reported as described in the original studies and are therefore not directly comparable across studies. IEX, ion exchange; ED, electrodialysis; MFC, microbial fuel cell; HCDI, hybrid capacitive deionisation; HTL, hydrothermal liquefaction; RO, reverse osmosis; FO, forward osmosis.

In hydroponic cultivation with liquid fertiliser (nutrient solutions), one key ingredient is potassium nitrate (KNO_3_) [82]. The required KNO_3_ content depends on the crop and growth stage, ranging from 7 to 59 kg m^−3^ in dependence of the type of solution [116]. The proportion of K in the fertiliser can be larger than the proportion of P. For example, lettuce needs 48 kg KNO_3_ in a special hydroponic fertiliser solution; however, P is included only in Monopotassium phosphate (KH_2_PO_4_), at 19 kg [116].

In general, solid fertiliser K-struvite with a molar ratio of 1:1:1 can be a good P fertiliser but, depending on the crop, a less sufficient K fertiliser. Furthermore, the results of Harada et al. [104] confirm that other struvite forms may also exist, which reduce the purity of K-struvite under real conditions.

Only one study, by Casadellà et al. [93], investigated how to produce a liquid KCl fertiliser (proof-of-concept with a single run; Section 3.4) using clinoptilolite-based mixed matrix membranes with synthetic and diluted urine, which confirms the research gap from Hofmann et al. [82]. Other liquid fertilisers include N–P–K fertiliser, and technologies like ED or FO mostly separate based on molecular weight and charge and are not yet selective for single nutrients. Moreover, other substances like micropollutants with similar molecular weight could also be separated in FO systems. Among the products reported, the aqueous by-product generated during HTL showed the highest K content in some experiments, showing potential as a K fertiliser [101]. For completeness, biochar as a fertiliser is listed in Table 5; although its K content is comparatively low, biochar may still provide value as an organic soil amendment.

Regarding fertilisers obtained from wastewater, only precipitated phosphate salts and their derivatives are currently approved as fertilisers under the EU Fertilising Product Regulation [117]. The regulation divides macronutrients into primary macronutrients (N, P, K) and secondary macronutrients (Ca, Mg, Na, Sulphur (S)). Based on this, the regulation defines product function categories (PFCs), for instance, inorganic fertiliser, as straight and compound fertiliser. A straight fertiliser contains only one macronutrient—regardless of whether it is primary or secondary—or only one primary macronutrient in combination with one or more secondary macronutrients. A compound fertiliser contains more than one primary macronutrient—regardless of whether they also contain one or more secondary macronutrients—or contains no primary macronutrient but more than one secondary macronutrient [117]. However, the Regulation promotes increased use of recycled nutrients when requirements are met and is currently being updated.

Because most studies remain at laboratory scale, the resulting fertilisers have not yet been approved or assessed under the EU Fertilising Product Regulation. Nevertheless, the categories into which the identified fertilisers can be classified are summarised in Table 6, if they were to fulfil the requirements. Regarding EU [117], only MPP, as a compound solid inorganic macronutrient fertiliser, is approved because it is a precipitated phosphate fertiliser.

**Table 6:** Potential product function categories (PFCs) and suitable wastewater streams for K fertilisers identified in the literature.

Fertiliser type	Fertiliser product	Assumed PFCs	Number of publications	Suitable wastewater streams
Liquid	KCl	Straight liquid inorganic macronutrient fertiliser	1	Effluent of wastewater treatment plants
Liquid	KOH in N–P–K solution	Compound liquid inorganic macronutrient fertiliser	1	Urine, livestock wastewater, and liquid digestate
Liquid	Cathodic solution with K, N, Na, Mg, and P	Compound liquid inorganic macronutrient fertiliser	1	Urine, livestock wastewater, and liquid digestate
Liquid	K_2_O in N–P–K mixed solution	Compound liquid inorganic macronutrient fertiliser	1	Urine, livestock wastewater, and liquid digestate
Liquid	N-P-K mixed solution	Compound liquid inorganic macronutrient fertiliser	1	Urine, livestock wastewater, and liquid digestate
Liquid	NH_4_^+^ and K solution in concentration stream	Compound liquid inorganic macronutrient fertiliser	1	Urine, livestock wastewater, and liquid digestate
Solid	Biochar	Organic soil improver	2	Urine
Solid	MPP	Compound solid inorganic macronutrient fertiliser	22	Urine, livestock wastewater, and liquid digestate

Note: MPP, magnesium potassium phosphate (K-struvite; KMgPO_4_·6H_2_O).

In the studies pollutant characterisation of the recovered fertiliser products was generally not reported (see Table 5). However, Winker et al. [118] provided an overview of potential hazards and risks from fertiliser products in new sanitation systems, while Wang et al. [119] showed the application of MPP on crops. Ciceri et al. [12] further proposed a multi-local potash market including substitutes, including those recovered from wastewater.

For classification purposes, EU regulations continue to specify quality requirements for fertilisers regarding the heavy metals cadmium, chromium, mercury, nickel, lead, arsenic, copper, and zinc. In addition, for organic fertilisers, *Salmonella* and *Escherichia coli* contamination must be tested and remain below the limit. The requirements for inorganic micronutrient fertiliser are shown in Supplementary Table S3. Furthermore, the regulation specifies the minimum macronutrient content, including the mass of K for each PFC. The requirements for PFCs shown in Table 6 by nutrients in mass (%) are displayed in the Supplementary Table S4.

Since previous investigations were predominantly conducted at laboratory scale to assess the fundamental suitability of various technologies for producing potassium fertilisers (proof of concept), only two investigations considered the above-mentioned aspects of pollutants with heavy metals [71], [120]. No study investigated effects regarding microorganisms. Furthermore, new contaminants such as organic micropollutants can become relevant, as wastewater contains these substances. Product purity must be considered more closely when scaling up processes. The probability of contamination varies by wastewater type; for example, urine likely contains no heavy metals but may contain organic contaminants.

The feasibility of producing a specific K fertiliser product also depends on the characteristics of the source wastewater. Based on current knowledge, it makes sense to recover compound fertiliser from nutrient-rich, highly concentrated streams (urine, process water). By contrast, K-based straight fertiliser should, where appropriate, be recovered from the effluent of the wastewater treatment plant, where K is present in a more favourable ratio compared to other nutrients.

A general methodology for comparing fertiliser content and application requirements should be considered alongside crop nutrient demand and the type of agriculture. Bijay-Singh and Sapkota [121] indicated that K-struvite is a slowly available K source for plants, which can be useful for traditional agriculture as a long-term field fertiliser. Hydroponics needs liquid fertiliser without the risk of precipitation, with concentrations adjusted for blooming or plant growth. Liquid fertiliser should be applied regionally because of transport costs. With a growing world population and a need for more circularity, different types of K-fertiliser products are needed, including liquid fertiliser for hydroponics for regional applications, fast-release fertilisers to compensate for nutrient deficiencies, and slow-release fertilisers for traditional agriculture. K fertilisers recovered from wastewater could be a good, versatile alternative to conventional fertilisers if they meet the requirements of the EU Fertilising Product Regulation. Because most research has been carried out at lab scale, no agronomic studies have been conducted to date. This must be considered as the technologies scale up.

At present, recycled K fertilisers for applications such as hydroponics are best understood as niche products, since there is no issue of finite supplies or increasingly severe contamination of raw material sources, unlike P fertilisers. However, the market for highly specialised hydroponic fertilisers (including specialised K fertiliser) is likely to grow and is already developing to varying degrees internationally, such as in Japan [122 123]. Furthermore, consumer focus may shift: in organic farming, MAP is the only mineral fertiliser permitted as an exception because it is a recycled product [117 124]. In the future, recycled potassium fertilisers could also compensate for deficiencies in organic farming.

Supplementary Fig. S1 provides an overview of the current state and potential K recovery options.

### 3.6 Future work and opportunities

When K-recovery technologies advance toward higher recovery efficiencies and greater technological maturity, future studies should include life cycle assessment (LCA) at increasing levels of readiness to assess the environmental impact and energy balance of different processes and products compared with traditional K mining.

LCA provides a systematic framework for evaluating environmental impacts throughout the life cycle of a product or process, including goal and scope definition, inventory analysis, impact assessment and interpretation [125]. The scope of an LCA for recycled products derived from wastewater should be carefully selected. At the outset, the system boundary should be clearly defined, for example, whether the assessment covers only the recovery stage and the recovered product or extends to the integration of the recovery stage along with the product within the entire wastewater treatment plant. The optimal LCA would be cradle-to-grave for the whole process. However, this requires a great deal of reliable data for the inventory phase. Based on the chosen scope, the inventory phase should capture all relevant inputs and outputs, including energy use, raw materials and physical inputs, waste streams, products and co-products such as pollution with heavy metals or organic micropollutants, emissions to air, water, or soil, transportation requirements, and other environmental aspects (e.g., eutrophication, transportation of the product to the end consumer).

To date, no studies have conducted an LCA specifically for K recovery from wastewater. Gahane and Mandavgane [126] provided an sustainability assessment of biogenic K recovery from biomass ash, demonstrating the potential value of LCA approaches for evaluating alternative K resources. Although their study differs from wastewater-based K recovery in terms of substrate type and technological scope, it highlights the importance of assessing the environmental performance of alternative K sources. Other studies, such as Abdolrezayi et al. [127], consider an LCA of P recovery from wastewater, which could benefit an initial assessment of K recovery and the application of the methodological approach.

As K recovery technologies progress beyond laboratory scale, LCA should be considered, with the necessary inventory data collected systematically. Early LCA integration can identify potential limitations, guide process optimisation, and support the development of sustainable wastewater-based K fertilisers.

## 4 Conclusion

K recovery from wastewater streams is an evolving area of research and has great potential for producing different kinds of sustainable fertilisers. Since, traditional K mining has high environmental impacts and food security demands are rising, suitable alternatives need to be developed.

The development of robust K recovery processes still needs ongoing research on validation under real wastewater conditions, up-scaling and feasibility of the technologies, implementation under real conditions into existing wastewater treatment plants and fertiliser quality. Furthermore, more research regarding long-term research on all K recovery technologies is necessary, highlighting another research gap. Favoured streams in K recovery research are currently urine, livestock manure, and liquid digestate because of their high concentrations (above 1000 mg L^−1^) whereby the full potassium potential in the wastewater cannot be utilized.

Most research on K recovery technologies obtained from wastewater is conducted under lab-scale or pilot-scale although the classification of whether an experiment is lab-scale or pilot-scale is described very vaguely and is defined inconsistently across publications. Clear categories would significantly facilitate the comparison of studies in the future.

All research topics of this review can be answered and discussed at this stage in the sections, except topic five concerning fertiliser quality (Section 1). A thorough response to topic five necessitates further research on the type and quality of potassium fertiliser sourced from wastewater. Since previous research has focused predominantly on MPP, other products -such as liquid or single-nutrient fertilisers - should be increasingly included in the research. In future research, recovery products should be explicitly defined from the outset, and potential contaminations should be examined in accordance with the EU Fertiliser Product Regulation and, additionally, for instance, organic micropollutants. The intended recycling product should always be considered when selecting wastewater streams and technologies, leaving plenty of room for further work.

The current status of potassium recovery described in the article should be progressively updated as future research progresses. New approaches which consider combinations of technologies to address challenges and achieve synergies should also be increasingly incorporated into research and also include suitable LCA for technology comparison and impact in longterm.

## CRediT authorship contribution statement

**Cora Eichholz:** Writing – original draft, Visualization, Validation, Software, Resources, Methodology, Investigation, Formal analysis, Data curation, Conceptualization. **Heidrun Steinmetz:** Writing – review & editing, Validation, Supervision, Project administration, Funding acquisition, Conceptualization.

## Data availability

The data presented in this study are available upon request from the corresponding author.

## Declaration of generative AI and AI-assisted technologies in the manuscript preparation process

During the literature research, the authors used Research Rabbit to extend the literature for the scoping review. After using this tool/service, the authors reviewed and edited the content as needed and take full responsibility for the publication's content.

During the manuscript preparation process, the authors used ChatGPT to improve language and readability. After using this tool/service, the authors reviewed and edited the content as needed and take full responsibility for the content of the published article.

## Declaration of competing interests

The authors declare that they have no known competing financial interests or personal relationships that could have appeared to influence the work reported in this paper.
