# Supplementary material for: Wastewater mining for circular potassium fertilisers: A critical scoping review of technologies, product formulations, and up-scaling challenges
Source: Environ Sci Ecotechnol. 2026 Oct 8;34:100777. doi: 10.1016/j.ese.2026.100777 (PMC13734989; doi:10.1016/j.ese.2026.100777)
Supplement: Supplementary file 1 [file mmc1.docx]

**Wastewater Mining for Circular Potassium Fertilisers: A Critical Scoping Review of Technologies, Product Formulations, and Up-scaling Challenges**

**SUPPLEMENTARY INFORMATION**

Cora Eichholz*, Heidrun Steinmetz

Department of Resource-Efficient Wastewater Treatment, RPTU Kaiserslautern-Landau, Paul-Ehrlich-Straße 14, 67663 Kaiserslautern, Germany

* Corresponding author:
Cora Eichholz ([cora.eichholz@rptu.de](mailto:cora.eichholz@rptu.de)).

[Text S1	Tree data extraction: Variables collected per paper	2](#_Toc242106218)

[Table S1	Overview of K, Na, Mg, Ca, P and N concentrations in different kinds of wastewater	5](#_Toc242106219)

[Table S2	Overview of different kinds of K recovery technologies due to type of ww, technology level, K recovery efficiency and molar ratio	7](#_Toc242106220)

[Table S3	Requirements of Fertiliser Quality for Inorganic Macronutrient Fertiliser	13](#_Toc242106221)

[Table S4	Requirements for PFCs: Nutrients by mass of [%]	14](#_Toc242106222)

[Figure S1	Overview optional ways of K recovery from ww due to ww streams, type of technology and type of fertiliser	15](#_Toc242106223)

[References	16](#_Toc242106224)

# **Text S1 Tree data extraction: Variables collected per paper**

In **Bold** categories (variables) given by author of this review based on research questions for data extraction. Underlined subcategories based on literature of this review:

- **Research Step for Scoping Review**
  - **Scopus**
  - **Research Rabbit**
    - **Earlier Work**
    - **Later Work**
- **Kind of paper**
  - Peer-Reviewed Research Article
  - Doctoral thesis
  - Book
  - Briefing
- **Origin of ww**
  - WWTP
    - Sludge
      - Dewatering sludge
      - Process water
      - Liquid digestates (after the application of anaerobic digestion)
      - Incinerated sewage sludge ash
      - Synthetic sewage sludge effluent
    - Secondary effluent
    - Primary settled wastewater
    - Municipal wastewater
      - Synthetic wastewater simulating municipal wastewater
  - Synthetic wastewater
  - Industrial wastewater
    - Semiconductor wastewater
      - Semiconductor sludge
    - Olive mill wastewater
      - Anaerobic digested olive mill wastewater
    - Landfill leachate concentrates
    - Gypsum pond wastewater
    - Brines
    - Vinasse
    - Water industries
    - Slaughterhouse wastewater
  - Livestock wastewater
    - Swine wastewater
    - Pig slurry
    - SISTRATES – Swine Wastewater Treatment System
    - Digestive slurry
    - Livestock ww after secondary treatment
    - Synthetic livestock ww
  - Blackwater
  - Faeces
  - Urine
    - Source-separated urine
    - Synthetic urine
    - Hydrolysed urine
    - Stored urine
    - Diluted urine
  - Waste streams in general
- **Technologies used for K recovery**
  - Ion exchange
  - Struvite precipitation
    - Fluidized-bed-reactor
    - Bubble column reactor with draught tube
  - Precipitation
  - Membrane filtration
    - Microfiltration
    - Nanofiltration
    - Ultrafiltration
    - Forward Osmosis
    - Reverse Osmosis
    - Coupled algae-osmosis-membrane system
    - Electrodialysis
    - Membrane Bioreactor
  - Biochar Production
    - Hydrothermal liquefaction
  - Microbial fuel cell
  - Bio-electro concentration
  - Evaporation
  - Distillation
  - Dehydration
  - Ozonation
  - Autotrophic nitrogen removal
  - Hybrid capacitive deionization
- **Technology level**
  - Lab scale
  - Pilot scale
  - Demo plant
  - Large scale/Full scale
  - Not defined
- **Recovery product**
  - Fertiliser
    - Liquid Fertiliser
      - Nutrient solution
    - Solid Fertiliser
      - K-Struvite
      - Biochar
      - Algae
  - Biocrude oil
  - Microalgae dewatering
  - No product
  - Fertigation / Water reuse

# **Table S1 Overview of K, Na, Mg, Ca, P and N concentrations in different kinds of wastewater**

Table 1: Concentration of K, Na, Mg, Ca, P and N (rounded) in different kinds of ww as a range and in average. ww after anaerobic digestion highlighted with asterisk. S.D. shows standard deviation. (*) … converted from mmol/L to mg/L. Due to N concentrations no uniform procedure has been found in the literature: (**) … was specified as NH_4_-N. (***) … was specified as Total Ammonium Nitrogen. (****) … was specified as NH_3_-N. (*****) … was specified as sum of NH_4_-N and NH_3_-N.

| *Type of ww* | *Subcategory type of ww* | *Total K* | | *Total Na* | | *Total Mg* | | *Total Ca* | | *Total P* | | *Total N* | | *Literature* |
| --- | --- | --- | --- | --- | --- | --- | --- | --- | --- | --- | --- | --- | --- | --- |
|  |  | *Range [mg/L]* | *Average ± S.D. [mg/L]* | *Range [mg/L]* | *Average ± S.D. [mg/L]* | *Range [mg/L]* | *Average ± S.D. [mg/L]* | *Range [mg/L]* | *Average ± S.D. [mg/L]* | *Range [mg/L]* | *Average ± S.D. [mg/L]* | *Range [mg/L]* | *Average ± S.D. [mg/L]* |  |
| *Urine* | Fresh | - | 3,382 ± 94 (*) | - | 3,610 ± 131 (*) | - | - | - | - | - | - | - | - | [1] |
|  | Fresh | - | 1,682 | - | 2,585 | - | 65 | - | 140 | - | 377 | - | 359 (**) | [2] |
|  | Fresh | - | 1,720 | - | 1,960 | - | - | - | - | - | 230 | - | 4,850 | [3] |
|  | Fresh | - | 1,470 | - | 1,620 | - | - | - | - | - | 169 | - | 3,820 (**) | [4] |
|  | Fresh | - | 1,947 ± 109 (*) | - | 6,851 ± 234 (*) | - | 1 ± 1 (*) | - | 3 (*) | - | 725 ± 34 (*) | - | 15 (*) | [5] |
|  | Hydrolysed |  | 946 (*) | - | 1,996 (*) | - | - | - | - | - | 492 (*) | - | 6,975 | [6] |
|  | Hydrolysed | - | 1,773 | - | 2,283 | - | 0 | - | 25 | - | 263 | - | 4,800 (*****) | [2] |
|  | Hydrolysed |  | 1,336 | - | 1,800 | - | 0 | - | 2 | - | - | - | 5,640 | [7] |
|  | Stored | - | 1,081 | - | 1,326 | - | - | - | - | - | 344 | - | 5,037 (***) | [8] |
|  | Stored |  | 1,410 ± 320 |  | 1,740 ± 360 |  | < 5 |  | 16 ± 3 |  | 208 ± 49 |  | 1,290 ± 250 (***) | [9] |
|  | Stored | 900-1,100 | - | 940-980 | - | 1-2 | - | 13-16 | - | 200-210 | - | 1,795-2,610 | - | [10] |
|  | Synthetic | - | 267 ± 59 | - | 625 ± 88 | - | 238 ± 41 | - | 81 ± 28 | - | 766 ± 11 | - | 472 ± 21 | [11] |
| *Livestock ww* | swine slurry (manure) | - | 757 ± 338 | - | - | - | - | - | - | - | 453 ± 368 | - | 1,221 ± 457 | [12] |
|  | pig slurry (manure) | - | 1,584 | - | 696 | - | 88 | - | 33 | - | 105 | - | 29 | [13] |
|  | pig manure |  | 1,600 ± 300 | - | - | - | - | - | - | - | - | - | - | [14] |
|  | cattle manure * | - | 1,000 | - | - | - | - | - | - | - | - | 800-1,200 | - | [15] |
|  | Cattle manure * (after membrane treatment) | - | 1,535 | - | 570 | - | 76 | - | 85 | - | 27 | - | 1,024 | [16] |
|  | Manure * | - | 892 ± 102 | - | 206 ± 81 | - | 31 ± 5 | - | 119 ± 15 | - | 25 ± 2 | - | 2,315 ± 41 | [17] |
|  | Synthetic livestock ww | - | 1,001 (*) | - | - | - | 2 (*) | - | - | - | 201 (*) | - | 78 (*) | [18] |
|  | Swine ww |  | 384 ± 13 |  | 118 ± 5 |  | 38 ± 2 |  | 42 ± 1 |  | 125 ± 8 |  | 465 ± 26 (***) | [19] |
| *Industrial ww* | Landfill leachate | 1,105 -2,141 | 1,933 ± 276 | 2,419-12,567 | 4,656 ± 3,118 | 59 - 115 | 77 ± 21 | 1-94 | 39 ± 28 | 0-7 | 7 ± 2 | 382-3,351 | 2,575 ± 891 | [20] |
|  | Olive mill ww * after centrifugation and filtration | - | 1,969 ± 10 | - | 796 ± 3 | - | - | - | - | - | 72 ± 1 | - | 14 | [21] |
|  | semiconductor ww | 9-49 |  | 6-40 | - | 1-2 | - | 6-12 | - | 5-388 | - | 80-250 | - | [22] |
|  | Vinasse |  | 1,390 ± 8 | - | 105 ± 3 | - | 178 ± 3 | - | 451 ± 10 | - | 98 ± 3 | - | 189 ± 6 (****) | [23] |
|  | Vinasse after ultrafiltration (UF) | - | 1,008 ± 36 | - | 71 ± 14 | - | 165 ± 9 | - | 408 ± 13 | - | 58 ± 17 | - | 78 ± 11 (***) | [24] |
| *ww streams at the WWTP* | Primary settled ww | - | 25 | - | 142 | - | 34 | - | 76 | - | 12 | - | 35 | [25] |
|  | Centrate | - | 251 ± 2 | - | - | - | 12 | - | 54 ± 1 | - | 59 ± 6 | - | 949 ± 80 | [26] |
|  | Liquid digestate * | 2,185-2,440 | - | 1,385-1,655 | - | 45-81 | - | 118-223 | - | 110-125 | - | 3,750-4,120 | - | [27] |
|  | Liquid digestate * | 1,494-2,337 | 1,981 | 1,268-1,505 | 1,399 | 22-105 | 54 | 41-697 | 298 | 271-549 | 450 | 1,787-3,409 | 2,557 | [28] |
|  | Liquid digestate * | - | 636 ± 20 | - | - | - | - | - | - | - | 356 ± 15 | - | 1,027 ± 30 (***) | [29] |
|  | Synthetic sewage sludge effluent |  | 113 | - | 137 | - | 21 | - | 14 | - | 50 | - | 880 | [30] |

# **Table S2 Overview of different kinds of K recovery technologies due to type of ww, technology level, K recovery efficiency and molar ratio**

Table 2: Overview of different kinds of K recovery technologies due to type of ww, technology level (scale), K recovery efficiency and molar ratio. Molar ratio refers to initial concentration. Scale refers to the technological level described in the publications. n.s. = not specified.

| *K recovery technology* | *Type of ww* | *Scale* | *K Recovery efficiency rounded [%]* | *Molar ratio* | *Further Details* | *Literature* |
| --- | --- | --- | --- | --- | --- | --- |
| *Wet-chemical processes (a)* | | | | | | |
| **K-Struvite precipitation** | Synthetic ww | lab | n.s. | Mg:K:P 3:50:1 | High K:P ratio of 45-50:1 to prevent precipitation products, no counter ions considered | [31] |
| **K-Struvite precipitation** | Pig slurry | lab | 25% | Mg:K:P 1.4:9.5:1 (denitrified effluent) 0.7:1.2:1.0 (waste sludge from bioreactor) | after nitrification. Problem with MAP crystallization | [13] |
| **K-Struvite precipitation** | Synthetic urine | lab | 59% | Mg:K:P  1:1:1 | Optimum pH = 11, considered counter ions: Na, NH_4_, Ca | [32] |
| **K-Struvite precipitation** | Synthetic urine | lab | 68% | P:Mg:N 1:1.4:1.2 | Mainly publication about struvite. pH = 9.5, considered counter ions: Na, Ca, NH_4_ | [11] |
| **K-Struvite precipitation** | Synthetic livestock ww | n.a. | n.a. | Mg:K:P 1:1:1 | Counter ions: Na, pH=11 | [33] |
| **K-Struvite precipitation** | Source-separated urine | lab | 70% | Mg:K:P 4:1:1.6 | due to stabilization agent | [5] |
| **K-Struvite precipitation** | Synthetic urine | lab | 50% for Mg(OH_2_) | Mg:K:P 1.5:1:1.6 - 5.5:1:1.6 | Pretreated by NH_4_ stripping. using different Mg sources. Counter ions: Na, Ca.  pH = 8.8-12.5 | [34] |
| **K-Struvite precipitation** | Stored urine | lab | 80% | Mg:P 1.1:1 (to ensure max TP recovery) | No molar ratio including K have been found in this study. | [8] |
| **K-Struvite precipitation** | Source-separated urine | full | 24% | Mg:P 1.0-2.2. | pH= 8.5-9.5. No molar ratio including K have been found in this study. | [35] |
| **K-Struvite precipitation** | Urine | lab | 71% | Mg:K ratio of 1.25 | pH=10, average crystal = 0.85 mm; purity = 95% | [36] |
| **K-Struvite precipitation** | Swine ww | lab | 74% | Mg:P ratio of 1.3 | pH = 9 | [19] |
| **K-Struvite precipitation** | Synthetic sewage sludge effluent | lab | n.s. | K:P over 3 necessary to form MPP | pH = 11.5, counter ions were considered | [30] |
| **K-Struvite precipitation** | Livestock ww after secondary treatment | lab | n.s. | Mg:K:P 1.0:2.5:8.5 | pH = around 10 | [37] |
| **K-Struvite precipitation** | Synthetic urine | lab | 35% + 15% (by adding prefabricated struvite crystals) | K:P to 1:0.6 | precipitation through electrochemical crystallization with a sacrificial Mg anode | [38] |
| **K-Struvite precipitation** | Synthetic ww | lab | > 90% | Mg:P 1:10, 1:3 and 1:1.5 (corresponding pH 9-11). Theoretical value Mg:K:P 1:1:1 | testing of various pH and initial Mg concentrations | [39] |
| **K-Struvite precipitation** | Source-separated urine (synthetic) | lab | n.s. | n.s. | competitive behaviour of MAP and MPP (KMP). pH around 9. Considering Na, Ca as counter ions. | [40] |
| **K-Struvite precipitation** | Synthetic urine | lab | 98% | Mg:K:P 2:1:2 | pH around 10. Considering Na, Ca as counter ions. | [41] |
| **K-Struvite precipitation** | Synthetic urine | lab | 76% | Mg:K:P 3.3:1:3 | laboratory-scale draft tube and baffle reactor. Mixing speed and hydraulic retention time are important operating factors | [42] |
| **K-Struvite precipitation** | Synthetic source-separated urine | pilot | 20-35% | Mg:P 1:1. | Fluidized-bed-reactor. pH = around 10.5. Counter ions considered: Na, NH_4_ and Ca. No molar ratio including K have been found in this study. | [43] |
| **K-Struvite precipitation** | Synthetic urine | lab | n.s. | Mg:P 0.3-0.8. | Operated in a stirred reactor. Also, Na-struvite has been formed at Mg:P 0.8. pH = 8 – 11. No molar ratio including K and Na have been found in this study. | [44] |
| **K-Struvite precipitation** | Livestock ww | n.s. | n.s. | K:P ~ 1.0 | pH=11. Counter ions: NH_4_, NO_3_, SO_4_ | [18] |
| **K-Struvite precipitation** | Centrate | lab | n.s. | Mg:P 0.75-1.25:1 | Partial nitritation-anammox (NH_4_ removal) can favour MPP. . No molar ratio including K have been found in this study. | [45] |
| **K-Struvite precipitation** | Pig slurry | pilot | ≤ 15%-51% | Mg:K:P 1.4:9.5:1.0 K as limiting factor: 1.0:0.5:1.0 | pH = 9 – 12. Counter ions: Na | [14] |
| **IEX** | Brines | lab | 90% (batch experiment: 1M NH_4_Cl as eluent solution) | n.s. | Focus on Rb^+^, K^+^ as a counter ion. | [46] |
| **IEX** | Liquid digestate (after anaerobic digestion) | lab | 37-78% (adsorption) | n.s. | Material: clinoptilolite. No focus on desorption | [27] |
| **IEX** | Anaerobically digested olive mill ww | lab | n.s. | n.s. | Adsorption and desorption process | [21] |
| **IEX** | Diluted urine, hydrolysed urine | lab | n.s. | n.s. | Material: Natural zeolites. Higher K adsorption to chabazite than clinoptilolite | [7] |
| **IEX** | Synthetic ww | n.s. | 100% regeneration (Moles eluted/ moles adsorbed) for synthetic resins, 43% clinoptilolite, biochar over 100% | n.s. | Adsorption Material: clinoptilolite, Biochar, Dowex 50 and Dowex Mac 3. Counter ions: NH_4_, Na. One cycle as a proof-of-concept | [4] |
| **IEX** | Hydrolysed urine | lab | 91% for Na-chabazite for best performing and chosen for further testing | n.s. | Adsorbents tested: Sludge biochar, maple biochar, Canada natural clinoptilolite, Iran natural clinoptilolite, Cuba natural clinoptilolite, Zeolite 4A China, Zeolite 4A Sigma, Na-chabazite, Ca/Na-Chabazite | [47] |
| **IEX** | Synthetic urine | pilot | 100-87% | n.s. | Dowex Mac 3 higher affinity towards NH_4_. Tests of different regenerants. Counter ions: NH_4_ | [48] |
| **IEX** | Landfill leachate concentrates | lab | 48% | n.s. | adsorption using natural zeolite | [20] |
| *Electrochemical processes (b)* | | | | | | |
| **HCDI** | Synthetic ww | lab | 36% (removal from ww) | n.s. | K reduction at 0.1 V | [49] |
| **MFC** | Swine ww | lab | 44-49% | n.s. | - | [50] |
| **ED** | Digestive slurry | lab | 70% at 7 Volt | n.s. | synchronously cationic and anionic selective electrodialysis (SCAE) | [17] |
| **ED** | Hydrolysed urine | lab | 50% | n.s. | Q = 133 mL/d with catholyte recirculation | [51] |
| **ED to form K-Struvite** | Vinasse | pilot | 68% | Mg:K 1.5-4.2:1 | - | [24] |
| **ED to form K-Struvite** | Vinasse | lab | >70% | n.s. | Tests of different ED configurations | [23] |
| **ED** | Urine | lab, pilot | 95% | n.s. | Best result at 20 Volt and a Flow rate of 0.1 mg/L | [52] |
| **ED** | Swine ww | lab | 13% (at 6 Volt) | n.s. | - | [53] |
| *Thermochemical processes (c)* | | | | | | |
| **HTL** | Synthetic secondary effluent irrigated willow | lab | 95% | n.s. | recovery in the aqueous phase as a byproduct from HTL | [54] |
| *Thermic processes (d)* | | | | | | |
| **Evaporation** | Urine | lab | 6% with sulphuric acid | n.s. | cycles of fresh urine addition and dehydration | [55] |
| **Evaporation** | Urine | lab | 72% | n.s. | To a concentration factor 30 (96.7% mass reduction) | [56] |
| **Distillation** | Source-separated urine | lab | 100% | n.s. | - | [9] |
| **Alkaline dehydration** | Stabilized urine | lab | 100% | n.s. | stabilization through alkaline media (wood ash or alkalised biochar) | [57] |
| **Alkaline dehydration** | Urine | lab | 8% at 60°C for Ash | n.s. | dehydration on different drying media: Bran-Ash, Char-Lime, Soil-Lime, Lime and Ash | [58] |
| *Physical processes (e)* | | | | | | |
| **FO** | Synthetic ww | pilot | n.s. | n.s. | FDFO = fertiliser drawn forward osmosis for hydroponic uses. Counter ions considered. | [59] |
| **FO** | Pig slurry | lab | 83% K_2_O | n.s. | due to alkaline treatment | [60] |
| **FO** | Urine | lab | n.s. | n.s. | as draw solution for algae | [61] |
| **FO** | Source-separated urine | lab | 79-97% | n.s. | tested for synthetic fresh urine, hydrolysed stale urine and treated stale urine | [62] |
| **RO** | Liquid digestate (after anaerobic digestion) | pilot | over 90% rejection | n.s. | - | [28] |
| *Combined processes* | | | | | | |
| **ED-FO** | Liquid digestate (after anaerobic digestion) | lab | 86% | n.s. | Best results for removal and recovery at a concentration factor 3.6 | [29] |
| **IEX/MMM** | Diluted urine | n.s. | 60% | n.s. | 30 wt% polymer and 70 wt% clinoptilolite. Desorption with hot water 60°C | [1] |

# **Table S3 Requirements of Fertiliser Quality for Inorganic Macronutrient Fertiliser**

Table 3: Requirements of Fertiliser Quality for Inorganic Macronutrient Fertiliser due to 63 [63]

| Contaminants | Fertiliser Quality | |
| --- | --- | --- |
|  | Inorganic Macronutrient Fertiliser | Note: |
| Cadmium (Cd) | 3 mg/kg dry matter | Note, where an inorganic macronutrient fertiliser has a total phosphorus (P) content of less than 5% phosphorus pentoxide (P_2_O_5_)-equivalent by mass |
|  | 60 mg/kg phosphorus pentoxide (P_2_O_5_) | Note, where an inorganic macronutrient fertiliser has a total phosphorus (P) content of 5% phosphorus pentoxide (P_2_O_5_)-equivalent or more by mass ('phosphate fertiliser') |
| Hexavalent chromium (Cr VI) | 2 mg/kg dry matter | - |
| Mercury (Hg) | 1 mg/kg dry matter | - |
| Nickel (Ni) | 100 mg/kg dry matter | - |
| Lead (Pb) | 120 mg/kg dry matter | - |
| Arsenic (As) | 40 mg/kg dry matter | - |
| Biuret (C_2_H_5_N_3_O_2_) | 12 g/kg dry matter | - |
| Perchlorate (ClO_4_^-^) | 50 mg/kg dry matter | - |
| Copper (Cu) | 600 mg/kg dry matter | This value shall not apply where Cu has been intentionally added to an inorganic macronutrient fertiliser for the purpose of correcting a soil micronutrient deficiency and is declared in accordance with Annex III. |
| Zinc (Zn) | 1500 mg/kg dry matter | This value shall not apply where Zn has been intentionally added to an inorganic macronutrient fertiliser for the purpose of correcting a soil micronutrient deficiency and is declared in accordance with Annex III. |

# **Table S4 Requirements for PFCs: Nutrients by mass of [%]**

Table 4: Requirements for PFCs due to 63 [63]: Nutrients by mass of [%]

| PFCs | | by mass of [%] | | | | | | |
| --- | --- | --- | --- | --- | --- | --- | --- | --- |
|  |  | N | P_2_O_5_ | K_2_O | MgO | CaO | SO_3_ | Na_2_O |
| Compound solid inorganic macronutrient fertiliser (Note 1 und 3) | - | 3 | 3 | 3 | 1.5 | 1.5 | 1.5 | 1 (Note 2) |
| Straight liquid inorganic macronutrient fertiliser | contains only one declared macronutrient | 5 | 5 | 3 | 2 | 6 | 5 | 1 (Note 2) |
|  | contains one primary macronutrient and one or more declared secondary macronutrients (Note 5) | 1.5  OR | 1.5 | 1.5  OR | 0.75 | 0.75 | 0.75 | 0.5 (Note 4) |
| Compound liquid inorganic macronutrient fertiliser (Note 5) | contains more than one primary macronutrient | 1.5 | 1.5 | 1.5 | 0.75 | 0.75 | 0.75 | 0.5 (Note 4) |
|  | contains more than one secondary macronutrient and no primary macronutrient |  |  |  |  |  |  |  |
| Organic soil improver | - | No further guidelines | | | | | | |

Note 1: A compound solid inorganic macronutrient fertiliser shall contain more than one of the following declared macronutrients in at least those contents.

Note 2: The total sodium oxide (Na_2_O) content shall not exceed 40% by mass.

Note 3: The sum of all declared macronutrient contents shall be at least 18% by mass.

Note 4: The total sodium oxide (Na_2_O) content shall not exceed 20% by mass.

Note 5: The sum of all declared macronutrient contents shall be at least 7% by mass.

**Figure S1	Overview optional ways of K recovery from ww due to ww streams, type of technology and type of fertiliser**

Figure 1: Overview shows optional ways of K recovery from ww (main findings of different chapters) due to ww streams, type of technology including technology stage (in brackets: l=lab-scale, p=pilot-scale, f=full-scale) and type of fertiliser. Orange streams are resource oriented. Purple streams are different stages at the WWTP. Green streams are agricultural, and livestock related. Blue streams are industrial originated. For feces no investigations have been found, which is highlighted with a cross.

References

[1]	A. Casadellà, P. Kuntke, O. Schaetzle et al., “Clinoptilolite-based mixed matrix membranes for the selective recovery of potassium and ammonium,” *Water research*, vol. 90, pp. 62–70, 2016.

[2]	T. L. Chipako and D. G. Randall, “Urine treatment technologies and the importance of pH,” *Journal of Environmental Chemical Engineering*, vol. 8, no. 1, p. 103622, 2020.

[3]	W. Pronk, M. Biebow, and M. Boller, “Electrodialysis for recovering salts from a urine solution containing micropollutants,” *Environmental science & technology*, vol. 40, no. 7, pp. 2414–2420, 2006.

[4]	W. A. Tarpeh, K. M. Udert, and K. L. Nelson, “Comparing Ion Exchange Adsorbents for Nitrogen Recovery from Source-Separated Urine,” *Environmental science & technology*, vol. 51, no. 4, pp. 2373–2381, 2017.

[5]	H. Huang, J. Li, B. Li et al., “Comparison of different K-struvite crystallization processes for simultaneous potassium and phosphate recovery from source-separated urine,” *The Science of the total environment*, vol. 651, Pt 1, pp. 787–795, 2019.

[6]	J. Liu, M. Zheng, C. Wang et al., “A green method for the simultaneous recovery of phosphate and potassium from hydrolyzed urine as value-added fertilizer using wood waste,” *Resources, Conservation and Recycling*, vol. 157, p. 104793, 2020.

[7]	U. Regmi and T. H. Boyer, “Ammonium and potassium removal from undiluted and diluted hydrolyzed urine using natural zeolites,” *Chemosphere*, vol. 268, p. 128849, 2021.

[8]	N. Jagtap and T. H. Boyer, “Integrated, multi-process approach to total nutrient recovery from stored urine,” *Environmental Science: Water Research & Technology*, vol. 4, no. 10, pp. 1639–1650, 2018.

[9]	K. M. Udert and M. Wächter, “Complete nutrient recovery from source-separated urine by nitrification and distillation,” *Water research*, vol. 46, no. 2, pp. 453–464, 2012.

[10]	H. Kirchmann and S. Pettersson, “Human urine - Chemical composition and fertilizer use efficiency,” *Fertilizer Research*, vol. 40, no. 2, pp. 149–154, 1995.

[11]	Q. Guan, G. Zeng, J. Song et al., “Highly efficient phosphorus and potassium recovery from urine via crystallization process in a fluidized bed reactor system,” *Journal of Environmental Chemical Engineering*, vol. 9, no. 4, p. 105623, 2021.

[12]	D. Cândido, A. C. Bolsan, C. E. Hollas et al., “Integration of swine manure anaerobic digestion and digestate nutrients removal/recovery under a circular economy concept,” *Journal of environmental management*, vol. 301, p. 113825, 2022.

[13]	E. Company, M. Farrés, J. Colprim et al., “Exploring the recovery of potassium-rich struvite after a nitrification-denitrification process in pig slurry treatment,” *The Science of the total environment*, vol. 847, p. 157574, 2022.

[14]	A. Magrí, E. Company, M. Farrès et al., “Recovery of Potassium-Rich Struvite in a Pig Farm Downstream a Nitrogen Removal Treatment Plant: Technological, Agricultural and Economic Assessment,” 2024.

[15]	X. Guo, L. Zeng, and X. Jin, “Advanced regeneration and fixed-bed study of ammonium and potassium removal from anaerobic digested wastewater by natural zeolite,” *Journal of environmental sciences (China)*, vol. 25, no. 5, pp. 954–961, 2013.

[16]	X. Guo, L. Le Zeng, X. Li et al., “Ammonium and potassium removal for anaerobically digested wastewater using natural clinoptilolite followed by membrane pretreatment,” *Journal of hazardous materials*, vol. 151, no. 1, pp. 125–133, 2008.

[17]	Y. Li, Z.-L. Ye, R. Yang et al., “Synchronously recovering different nutrient ions from wastewater by using selective electrodialysis,” *Water science and technology : a journal of the International Association on Water Pollution Research*, vol. 86, no. 10, pp. 2627–2641, 2022.

[18]	H. Harada, Y. Katayama, A. Afriliana et al., “Effects of Co-Existing Ions on the Phosphorus Potassium Ratio of the Precipitate Formed in the Potassium Phosphate Crystallization Process,” *Journal of Environmental Protection*, vol. 08, no. 11, pp. 1424–1434, 2017.

[19]	G. van Le, T. A. Luu, N. T. Bui et al., “Fluidized–bed homogeneous granulation for potassium and phosphorus recovery: K-struvite release kinetics and economic analysis,” *Journal of the Taiwan Institute of Chemical Engineers*, vol. 139, p. 104494, 2022.

[20]	P. Alirezazad, M. Fazeli Sangani, M. Khalili Rad et al., “Integrated treatment approach for recovering nutrients and reducing pollution load of landfill leachate: Targets towards a circular economy,” 2025.

[21]	D. Mitrogiannis, M. Psychogiou, G. Manthos et al., “Phosphorus and potassium recovery from anaerobically digested olive mill wastewater using modified zeolite, fly ash and zeolitic fly ash: a comparative study,” *Journal of Chemical Technology & Biotechnology*, vol. 97, no. 7, pp. 1860–1873, 2022.

[22]	H.-D. Ryu, C.-S. Lim, M.-K. Kang et al., “Evaluation of struvite obtained from semiconductor wastewater as a fertilizer in cultivating Chinese cabbage,” *Journal of hazardous materials*, 221-222, pp. 248–255, 2012.

[23]	A. Flávia Rezende Silva, L. A. Ribeiro, and M. C. S. Amaral, “Efficiency of nutrients recovery from sugarcane vinasse treatment by different electrodialysis configurations and in sequential-batch operation,” *Separation and Purification Technology*, vol. 311, p. 123295, 2023.

[24]	A. Flávia Rezende Silva, Y. A. R. Lebron, Y. L. Brasil et al., “Effect of electrolyte solution recycling on the potassium recovery from vinasse by integrated electrodialysis and K-struvite precipitation processes,” *Chemical Engineering Journal*, vol. 450, p. 137975, 2022.

[25]	W. Jiang, L. Lin, S. M. H. Gedara et al., “Potable-quality water recovery from primary effluent through a coupled algal-osmosis membrane system,” *Chemosphere*, vol. 240, p. 124883, 2020.

[26]	S. Johansson, M. Ruscalleda, B. Saerens et al., “Potassium recovery from centrate: taking advantage of autotrophic nitrogen removal for multi‐nutrient recovery,” *Journal of Chemical Technology & Biotechnology*, vol. 94, no. 3, pp. 819–828, 2019.

[27]	N. P. Kocatürk‐Schumacher, S. Bruun, K. Zwart et al., “Nutrient Recovery From the Liquid Fraction of Digestate by Clinoptilolite,” *CLEAN – Soil, Air, Water*, vol. 45, no. 6, 2017.

[28]	V. Proskynitopoulou, I. Garagounis, A. Vourros et al., “Nutrient recovery from digestate: Pilot test experiments,” *Journal of environmental management*, vol. 353, p. 120166, 2024.

[29]	Q. Tran, M. Garcia-Jaramillo, J. Schindler et al., “Sustainable nutrient water recovery by a hybrid electrodialysis (ED) - forward osmosis (FO) process for agricultural application,” *Journal of Environmental Chemical Engineering*, vol. 12, no. 2, p. 112091, 2024.

[30]	S. Nakao, T. Nishio, and Y. Kanjo, “Simultaneous recovery of phosphorus and potassium as magnesium potassium phosphate from synthetic sewage sludge effluent,” *Environmental technology*, vol. 38, no. 19, pp. 2416–2426, 2017.

[31]	A. M. Bennett, S. Lobanov, F. A. Koch et al., “Improving potassium recovery with new solubility product values for K-struvite,” *Journal of Environmental Engineering and Science*, vol. 12, no. 4, pp. 93–103, 2017.

[32]	Y. Gao, B. Liang, H. Chen et al., “An experimental study on the recovery of potassium (K) and phosphorous (P) from synthetic urine by crystallization of magnesium potassium phosphate,” *Chemical Engineering Journal*, vol. 337, pp. 19–29, 2018.

[33]	E. Hidayat and H. Harada, “Simultaneously Recovery of Phosphorus and Potassium Using Bubble Column Reactor as Struvite-K and Implementation on Crop Growth,” in *Crystallization and Applications,* Y. Ben Smida and R. Marzouki, Eds., IntechOpen, 2022.

[34]	H. Huang, D. Zhang, W. Wang et al., “Alleviating Na+ effect on phosphate and potassium recovery from synthetic urine by K-struvite crystallization using different magnesium sources,” *The Science of the total environment*, vol. 655, pp. 211–219, 2019.

[35]	Z. S.-G. Jang, K. Lim, and H. Leverenz, “Modulation of struvite composition in full-scale nutrient recovery system using source-separated urine,” *Water science and technology : a journal of the International Association on Water Pollution Research*, vol. 88, no. 4, pp. 1074–1086, 2023.

[36]	V.-G. Le, C.-T. Vu, Y.-J. Shih et al., “Phosphorus and potassium recovery from human urine using a fluidized bed homogeneous crystallization (FBHC) process,” *Chemical Engineering Journal*, vol. 384, p. 123282, 2020.

[37]	Y. Satoshi, O. Seichiro, H. Hiroyuki et al., “Simultaneous crystallization of phosphate and potassium as magnesium potassium phosphate using bubble column reactor with draught tube,” *Journal of Environmental Chemical Engineering*, vol. 1, no. 4, pp. 1154–1158, 2013.

[38]	J. Shan, H. Liu, S. Long et al., “Electrochemical crystallization for recovery of phosphorus and potassium from urine as K-struvite with a sacrificial magnesium anode,” *Environmental Chemistry Letters*, vol. 20, no. 1, pp. 27–33, 2022.

[39]	L. Wei, T. Zhang, T. Hong et al., “Revealing and quantifying the effect of cattiite coprecipitation on the purity of K-struvite in aqueous solution,” *Journal of Environmental Chemical Engineering*, vol. 11, no. 3, p. 109764, 2023.

[40]	J. A. Wilsenach, C. A. H. Schuurbiers, and M. C. M. van Loosdrecht, “Phosphate and potassium recovery from source separated urine through struvite precipitation,” *Water research*, vol. 41, no. 2, pp. 458–466, 2007.

[41]	K. Xu, C. Wang, H. Liu et al., “Simultaneous removal of phosphorus and potassium from synthetic urine through the precipitation of magnesium potassium phosphate hexahydrate,” *Chemosphere*, vol. 84, no. 2, pp. 207–212, 2011.

[42]	K. Xu, C. Wang, X. Wang et al., “Laboratory experiments on simultaneous removal of K and P from synthetic and real urine for nutrient recycle by crystallization of magnesium-potassium-phosphate-hexahydrate in a draft tube and baffle reactor,” *Chemosphere*, vol. 88, no. 2, pp. 219–223, 2012.

[43]	C. Zhang, K. Xu, J. Li et al., “Recovery of Phosphorus and Potassium from Source-Separated Urine Using a Fluidized Bed Reactor: Optimization Operation and Mechanism Modeling,” *Industrial & Engineering Chemistry Research*, vol. 56, no. 11, pp. 3033–3039, 2017.

[44]	C. Zhang, K. Xu, M. Zheng et al., “Factors Affecting the Crystal Size of Struvite-K Formed in Synthetic Urine Using a Stirred Reactor,” *Industrial & Engineering Chemistry Research*, vol. 57, no. 50, pp. 17301–17309, 2018.

[45]	S. Johansson, *Taking advantage of autotrophic nitrogen removal: Potassium and phosphorus recovery from municipal wastewater,* Doctoral thesis, University of Girona, 2018.

[46]	C. Dagan-Jaldety, P. Nativ, Y. S. Cristal et al., “A Prussian-blue analogue (PBA) ion-chromatography-based technique for selective separation of Rb+ (as RbCl) from brines,” *Water research*, vol. 247, p. 120757, 2023.

[47]	H. Wu, X. Foster, H. Kazemian et al., “N, P, K recovery from hydrolysed urine by Na-chabazite adsorption integrated with ammonia stripping and (K-)struvite precipitation,” *The Science of the total environment*, vol. 857, Pt 1, p. 159277, 2023.

[48]	W. A. Tarpeh, I. Wald, M. Wiprächtiger et al., “Effects of operating and design parameters on ion exchange columns for nutrient recovery from urine,” *Environmental Science: Water Research & Technology*, vol. 4, no. 6, pp. 828–838, 2018.

[49]	X. Zhao, D. Li, L. Deng et al., “Enhanced hybrid capacitive performance for efficient and selective potassium extraction from wastewater: Insights from regulating electrode potential,” 2025.

[50]	M. Gualtieri, A. Goglio, E. Clagnan et al., “The importance of the electron acceptor: Comparison between flooded and tidal bioelectrochemical systems for wastewater treatment and nutrients enriched solution production,” *Bioresource Technology Reports*, vol. 24, p. 101617, 2023.

[51]	J. Monetti, P. Ledezma, and S. Freguia, “Optimised operational parameters for improved nutrient recovery from hydrolysed urine by bio-electroconcentration,” *Separation and Purification Technology*, vol. 279, p. 119793, 2021.

[52]	H.-R. Yang, Y. Liu, S.-J. Hu et al., “Advanced electrochemical membrane technologies for near-complete resource recovery and zero-discharge of urine: Performance optimization and evaluation,” 2024.

[53]	Z.-L. Ye, K. Ghyselbrecht, A. Monballiu et al., “Fractionating various nutrient ions for resource recovery from swine wastewater using simultaneous anionic and cationic selective-electrodialysis,” *Water research*, vol. 160, pp. 424–434, 2019.

[54]	F. Conti, S. S. Toor, T. H. Pedersen et al., “Biocrude production and nutrients recovery through hydrothermal liquefaction of wastewater irrigated willow,” *Biomass and Bioenergy*, vol. 118, pp. 24–31, 2018.

[55]	P. Simha, A. Vasiljev, D. G. Randall et al., “Factors influencing the recovery of organic nitrogen from fresh human urine dosed with organic/inorganic acids and concentrated by evaporation in ambient conditions,” *The Science of the total environment*, vol. 879, p. 163053, 2023.

[56]	Y. Gao, B. Vinnerås, and P. Simha, “Partitioning behavior and crystallization of urea, salts and water during stepwise dehydration of acidified human urine by evaporation,” 2025.

[57]	P. Simha, J. Senecal, A. Nordin et al., “Alkaline dehydration of anion-exchanged human urine: Volume reduction, nutrient recovery and process optimisation,” *Water research*, vol. 142, pp. 325–336, 2018.

[58]	P. Simha, C. Lalander, A. Nordin et al., “Alkaline dehydration of source-separated fresh human urine: Preliminary insights into using different dehydration temperature and media,” *The Science of the total environment*, vol. 733, p. 139313, 2020.

[59]	L. Chekli, J. E. Kim, I. El Saliby et al., “Fertilizer drawn forward osmosis process for sustainable water reuse to grow hydroponic lettuce using commercial nutrient solution,” *Separation and Purification Technology*, vol. 181, pp. 18–28, 2017.

[60]	R. Rodríguez-Alegre, J. Zapata-Jiménez, X. You et al., “Nutrient recovery and valorisation from pig slurry liquid fraction with membrane technologies,” *The Science of the total environment*, vol. 874, p. 162548, 2023.

[61]	F. Volpin, H. Yu, J. Cho et al., “Human urine as a forward osmosis draw solution for the application of microalgae dewatering,” *Journal of hazardous materials*, vol. 378, p. 120724, 2019.

[62]	J. Zhang, Q. She, V. W. C. Chang et al., “Mining nutrients (N, K, P) from urban source-separated urine by forward osmosis dewatering,” *Environmental science & technology*, vol. 48, no. 6, pp. 3386–3394, 2014.

[63]	EU, *Regulation (EU) 2019/ of the European Parliament and of the Council of 5 June 2019 laying down rules on the making available on the market of EU fertilising products and amending Regulations (EC) No 1069/2009 and (EC) No 1107/2009 and repealing Regulation (EC) No 2003/2003*, 2019.
